# A Learning-Guided Meta-Heuristic Approach for Task Offloading in Four-Tier IoT Networks: A Hybrid UCB-ACO Algorithm

**DOI:** 10.3390/biomimetics11070509

**Published:** 2026-07-20

**Authors:** Lütfiye Özlem Akkan

**Affiliations:** İzmir Vocational School, Dokuz Eylul University, Izmir 35380, Turkey; ozlem.karaca@deu.edu.tr; Tel.: +90-232-301-2599

**Keywords:** computation offloading, mobile edge computing, ant colony optimization, upper confidence bound, UCB-ACO hybrid algorithm, energy efficiency, multi-objective optimization

## Abstract

The rapid development and expansion of the Internet of Things (IoT) ecosystem require managing increasing computational demands with the aid of advanced hierarchical architectures. The integration of a complete four-tier hierarchy—including Mobile, Edge, Fog, and Cloud layers—and the priority requirements are being overlooked in the literature despite the effort of existing studies. The goal of this study is to fill these gaps by proposing a novel, context-aware task-offloading framework designed for multi-dimensional ecosystems involving multi-server and multi-application environments. A targeted biomimetic approach is utilized at the core of this research. The decentralized foraging behavior of biological swarms is translated into a concrete engineering solution. This solution is designed specifically for computational offloading and resource management. To achieve this, a “Learning-guided Meta-heuristic” hybrid model is developed. Within this framework, bio-inspired Ant Colony Optimization (ACO) is directly integrated with an Upper Confidence Bound (UCB)-inspired exploration mechanism. Natural, pheromone-based imitation is solely relied upon by traditional biomimetic algorithms. In contrast, higher-order cognitive learning is fully incorporated by this hybrid synergy. Consequently, underlying system dynamics are adaptively learned. Local minima traps are also successfully avoided. This avoidance is achieved by dynamically selecting the optimal layer for each individual task. Both energy consumption and latency are optimized simultaneously. Meanwhile, strict operational feasibility is ensured through a dynamic penalty-based mechanism. Battery and deadline constraints are explicitly handled by this mechanism. Extensive simulations demonstrate the superiority of the proposed UCB-ACO model over state-of-the-art meta-heuristics, including Particle Swarm Optimization (PSO), Gray Wolf Optimizer (GWO), ACO, Artificial Bee Colony Optimization (ABO), and Non-dominated Sorting Genetic Algorithm II (NSGA-II). The findings reveal that the proposed framework outperforms the methods compared by achieving 22.5% lower latency and 23% lower energy consumption. This study effectively maps the current literature and then introduces a pioneering solution for next-generation resource management in distributed computing.

## 1. Introduction

Cloud computing is at the center of today’s digital transformation and modern IT ecosystem, initiating a revolutionary paradigm shift in data processing, storage, and management. As Internet of Things (IoT) technologies spread rapidly, handling the data generated by billions of Edge devices has become increasingly difficult with traditional infrastructure. Real-time execution of complex algorithms and big data analytics are possible using the high computing power, scalable resource management, and cost-effectiveness of Cloud computing. This technology is not only simple data storage, but also a critical foundation for the sustainability and accessibility of systems in various fields, including smart cities and autonomous systems.

End devices in IoT-based networks, such as smart home systems, generally face hardware bottlenecks, such as limited battery life, low processing capacity, and limited memory. These limitations made operating modern, locally based applications that require high-level computing difficult. The expansion of the IoT ecosystem has made the model of processing data only on central Cloud servers inadequate due to latency and bandwidth constraints. Hierarchical computing architectures, developed to overcome these bottlenecks and limitations, bring computational power closer to the data source by involving Edge and Fog layers into the process. Here, the Edge layer enables real-time, low-latency processing on the Edge devices where the data is generated or on local nodes located very close to them; the Fog layer serves as a strategic link between these two endpoints, offering a wider coverage area and moderate resource capacity. The addition of the Cloud layer to the three-layer (Edge–Fog–Cloud) architecture enhances the scalability of modern networks. However, the heterogeneous resource structure and varying energy characteristics of each layer make system management a complex optimization problem.

At this point, the task-offloading mechanism serves as a critical solution to the system’s optimization problem, in which the end devices have limited energy and processing capacity. This mechanism involves strategically transferring the heavy calculation workload from local devices to powerful Cloud or Edge servers. Efficient task-offloading management under dynamic and fluctuating network conditions has recently become a primary area of research due to the need to balance energy efficiency and computing speed.

Although many studies focus on either Cloud computing or Edge computing alone, the real challenge involves selecting the appropriate choice between the Edge and Fog layers to meet application needs (e.g., latency sensitivity, data size, and energy consumption). This intelligent layer selection is performed under dynamic network conditions, and it extends the system’s total lifespan while also improving quality of service (QoS) by reducing latency.

The current approaches in the mentioned literature usually address the trade-off between energy and latency by using static threshold values or single-layer architectures. However, in modern IoT ecosystems with a heterogeneous task structure, the requirements of each task and the network’s current load status change dynamically. This situation means that solutions focused only on the Cloud or only on Edge computing are insufficient for requirements.

Despite the significant advancements in IoT resource management, achieving a seamless balance between energy efficiency and latency in multi-layered architecture remains a formidable challenge. Traditional task-offloading strategies tend to prioritize a single performance metric or assume a uniform application environment, which is seldom true in reality. This leaves a research gap, especially in terms of adaptive frameworks. While taking distributed networks into account, these frameworks can navigate the complex interplay between heterogeneous Edge and Fog layers.

In many ways, IoT and Edge–Fog–Cloud architectures mirror biological ecosystems in their highly decentralized, dynamic nature. In nature, organisms such as ant colonies or bee swarms operate without centralized control. Nevertheless, they flawlessly execute complex tasks like resource allocation, workload distribution, and survival under severe energy constraints. The goal of biomimetics, or bionics, is to translate these evolutionary optimization mechanisms into mathematical models to solve complex engineering problems. Consequently, swarm intelligence-based biomimetic algorithms, particularly Ant Colony Optimization (ACO), have emerged as a natural fit for multi-tiered network environments. Traditional deterministic methods simply collapse under the weight of NP-hard task-offloading challenges. To resolve this, bio-inspired computational models mimic the pheromone signaling. These models also mimic adaptive choices of biological ants, thus they provide a robust, decentralized alternative. However, mapping raw biology directly onto artificial networks reveals a clear bottleneck. Real IoT channels are highly volatile, with volatile fluctuations and stochastic latency. Standard biological pheromone buildup is simply too slow to handle these rapid network shifts.

This operational gap is directly bridged here. Traditional bionics is elevated into a specialized “cognitive biomimetic” framework. This transition is achieved by upgrading the classic ACO structure with a learning-guided mechanism. Crucially, basic, blind swarm intelligence is surpassed. Actual higher-order cognitive functions are mimicked instead. Specifically, historical interaction data is utilized to actively balance exploration and exploitation. Through this integration, algorithmic agents are enabled to adapt to sudden network fluctuations on the fly. Ultimately, the feasibility of mathematically evolving nature-inspired concepts is proven. The successful orchestration of complex, multi-dimensional computing ecosystems is thus achieved. To address these limitations, this study proposes a novel hybrid meta-heuristic framework that integrates the Upper Confidence Bound (UCB)-inspired exploration mechanism with Ant Colony Optimization (ACO). In the context of this architecture, the integration of UCB does not imply a strict statistical framework; it is an adaptive empirical tracking mechanism. Unlike standard optimization techniques, the proposed UCB-ACO model dynamically allocates tasks while simultaneously minimizing energy consumption and latency. This hybrid mechanism leverages ACO’s exploration capabilities and UCB’s selection efficiency for robust decision-making. With this, multi-layered offloading processes can be managed adaptively.

One of the primary contributions of this research is the granular performance assessment conducted across multiple application types, reflecting the diverse workload characteristics of modern IoT systems. The effectiveness of the UCB-ACO model is rigorously validated through extensive simulation scenarios. The model performance is compared against established benchmarks, including ACO, Particle Swarm Optimization (PSO), Gray Wolf Optimizer (GWO), Artificial Bee Colony Optimization (ABO), and Non-dominated Sorting Genetic Algorithm II (NSGA-II). The findings demonstrate that the proposed model significantly outperforms traditional methods. It has provided more balanced and scalable solutions, especially in discrete and multi-objective optimization environments.

This paper proposes an ACO algorithm that addresses the Edge–Fog–Cloud hierarchy in an integrated manner and is enhanced with a UCB-based exploration capability, with the aim of overcoming the aforementioned limitations. The main contributions of this study can be summarized as follows:

1. Proposing a multi-objective task-offloading model that simultaneously optimizes energy consumption and latency,
2. Developing a hybrid meta-heuristic algorithm capable of making dynamic decisions between the Edge and Fog layers,
3. Demonstrating the performance advantage of the proposed model over traditional methods through comprehensive simulations.
4. Conducting application-based comparisons across multiple application types to validate the proposed model’s effectiveness under diverse workload requirements.

The rest of the article is organized as follows: Section 2 presents relevant studies in the literature and statistical analyses; Section 3 details the proposed system model and mathematical framework. Section 4 presents the proposed optimization method, and Section 5 presents the experimental results. Section 6 discusses the findings, and Section 7 outlines future research objectives.

## 2. Related Work

In this section, the current state and solution approaches to the task-offloading problem in multi-tier network architectures are investigated from an integrated perspective. In heterogeneous structures consisting of mobile devices, Edge, Fog, and Cloud servers, the computational load created by multi-user scenarios and different application types (multi-applications) takes the problem to a complexity beyond traditional methods. Especially, the conflicting nature between minimizing energy consumption and optimizing latency makes the problem display a character of multi-objective and NP-hard. To present this complexity systematically, the literature review was categorized into three main areas. First, recent survey studies were summarized to identify general trends in the field; then, studies focusing on energy and latency were assessed. Second, given that these problems involve such trade-off mechanisms and are naturally complex, the types of optimization methods that should be selected—specifically meta-heuristic and bio-inspired optimization methods such as Non-dominated Sorting Genetic Algorithm II (NSGA-II) [1], Ant Colony Optimization ACO [2], Particle Swarm Optimization (PSO) [3], Artificial Bee Colony Optimization (ABO) [4], and Gray Wolf Optimizer (GWO) [5] which are used to scan this complex solution space—have been evaluated comparatively in terms of their performance and application areas as reported in the literature. Finally, the studies in the literature were examined on a comparative table to emphasize the differences and improvements in the proposed study.

### 2.1. Surveys

To establish a rigorous conceptual baseline for distributed resource management, comprehensive review studies published over the past five years have been systematically analyzed [6,7,8,9,10,11,12,13,14,15,16,17,18,19,20]. Rather than treating the prior literature in a purely narrative or qualitative manner, this subsection clusters recent survey findings into three sequential thematic groups. This approach highlights the architectural evolution of paradigms, intelligence-driven algorithmic distributions, and multi-objective metric quantifications across the Cloud–Edge continuum.

#### 2.1.1. Algorithmic Paradigms and Architecture in Fog–Cloud Topologies

There is a dramatic shift away from centralized Cloud infrastructures toward layered, highly heterogeneous computing nodes in the recent meta-analytical literature. In Fog computing networks, for example, structural mappings emphasize the need for a new generation of genetic algorithm topologies that navigate volatile resource heterogeneity [6]. A statistical analysis of the broader algorithmic distribution in this domain shows that 25% of workflow planning models use classical heuristics, but 75% use advanced artificial intelligence (AI)-driven or parametric modeling frameworks [7]. A comprehensive review of the scheduling literature found that 97% of evaluated studies formulate the problem as multi-objective, with empirical simulation environments being used as primary validation methods in 51% of these frameworks [8]. In order to solve these operational bottlenecks, overarching taxonomies have extensively mapped task scheduling and resource allocation boundaries across unified Fog, Edge, and hybrid architectures [9]. There is a lot of momentum here, as 31% of foundational survey publications are coming out within the last three years. Quantitative resource allocation metrics prioritize execution cost (28.7%), network latency (24.1%) and deterministic execution time (22.2%) [10].

#### 2.1.2. Latency-Sensitive Edge/MEC Topologies and Intelligent Frameworks

Optimization challenges for high-density, latency-sensitive applications where real-time computing and strict reliability are crucial can also be found in the literature. Researchers confirm latency, energy consumption, and cost are the tri-focal parameters in distributed orchestration, with latency being the most frequently addressed [11]. These studies also note that large-scale meta-heuristic deployments get slowed down by manual hyperparameter tuning [11]. A robust Edge AI infrastructure and specialized resource management frameworks are required to solve this problem [12]. Particularly since standard Cloud paradigms are not suitable for time-critical IoT loops due to backhaul propagation bottlenecks [13]. Meta-heuristics prove powerful scaling advantages when searching massive, unexplored solution spaces in multi-access Mobile Edge Computing (MEC) to circumvent these limitations [14]. Additionally, recent surveys highlight the urgent need for lightweight, adaptive, and context-aware confidence frameworks that enable real-time, intelligent decision-making in decentralized environments [15].

#### 2.1.3. Quantitative Metric Allocation and Multi-Objective Trade-Offs

Offloading objective functions are the topic of the third thematic cluster. An analysis of data offloading frameworks in industrial IoT environments shows that time-related criteria (e.g., delay) and energy usage account for 24% and 20% of the active literature, respectively [16]. Classifying these mechanisms into classical, heuristic, and AI-based models reveals that communication parameters have a non-negligible impact on latency and power consumption [17]. Soft computing frameworks emphasize that ignoring localized trade-offs between delay, energy, and cost leads to suboptimal solutions that severely impact the efficiency of global systems [18]. From an energy-centric perspective, specialized scheduling reviews report that energy parameters dictate 28% of data transfer optimization efforts, followed by time variables at 16% [19]. According to these reports, traditional meta-heuristics have slow convergence profiles and high computational complexity, so hybrid meta-heuristics and machine learning integration are the most viable [19]. Compared to recent quality of service (QoS) mappings, energy efficiency takes the top spot at 22.86%, delay takes the third spot, and optimization problems remain the top academic driver in modern research [20].

### 2.2. Energy- and Latency-Focused Studies

In task-offloading processes, energy consumption and end-to-end latency represent the two most critical parameters governing system performance. Literature reviews show that researchers prioritize divergent optimization objectives because of the inherent trade-off between these two metrics. For example, some approaches emphasize energy efficiency to make resource-constrained Edge nodes last longer, while others focus on low latency to satisfy real-time application requirements. In some studies, different metrics are given different priority weights; for instance, time and energy are given 24% and 20% priority, respectively [16]. Alternative findings show energy as the top priority at 28%, followed by time at 16% [19]. Also, latency (22%) and energy consumption (17%) remain the top two research challenges in MEC environments, as a wide-ranging analysis shows that latency and energy are the primary metrics of interest across 93 and 75 studies, respectively [17].

Delay, energy consumption, and cost are the main performance metrics, while delay is the most frequently evaluated [11]. A balanced trade-off that mitigates energy load while maintaining overall system performance can be achieved using multi-objective optimization techniques [20]. According to [8], 97 percent of studies use multi-objective formulations—like minimizing delays and SLA violations while meeting deadlines—while a negligible fraction (3%) consider single-objective approaches. In Cloud computing environments, metrics like makespan, resource utilization, load balancing, and cost have a high significance level above 50%, while in Fog computing, makespan, delay, energy consumption, execution time, and cost win.

In Fog computing, energy efficiency is a key criterion, since most strategies to minimize processing time are dependent on latency or energy savings [10]; similarly, traffic state optimization was modeled as a multi-objective problem to balance computational load, energy consumption, and communication latency [12]. To conserve energy, Edge devices often get slower when they are running less computationally; this simultaneous optimization of conflicting objectives requires complex compromises, making it hard to find a near-optimal task allocation strategy in heterogeneous and dynamic Cloud and Fog environments [7]. Consequently, computational task offloading is widely recognized as a multi-objective, NP-hard optimization problem that cannot be solved in polynomial time using classical deterministic methods [13].

An evaluation of proposed architectural frameworks in the literature reveals clear distinctions regarding their hierarchical depth and architectural scope. To maximize conflicting goals such as processing time (makespan) and energy consumption, some studies focus solely on “Cloud” computing [21]. This omits intermediate Fog and Edge layers which add complexities to the structure and network. A substantial portion of the literature structures task allocation across dual-layer paradigms, including Fog–Cloud [22,23], Edge–Cloud [24,25], or intricate Mixed Fog/Cloud infrastructures [26]. Specifically, Ref. [24] proposes a collaborative Edge and Cloud framework to solve IoT-based computational discharging problems. It uses three distinct Constrained Multi-Objective Evolutionary Algorithms (CMOEAs). Meanwhile, the J-NECORA framework presented in [27] targets industrial IoT scenarios and mobile nodes to address the Cloud–Edge–Things continuum. In a similar vein, Ref. [25] investigates task offloading within Edge–Cloud systems through the lens of pricing and deadline constraints. But Ref. [28] directly focuses on the Cloud–Fog interaction to achieve an optimal energy–delay balance.

To further enhance architectural depth, another approach leverages three-tier hierarchies spanning local, Fog, and Cloud layers [22]. Some approaches utilize multi-user MEC networks covering mobile device (MD), Edge, and Cloud layers [29,30]. Among these hierarchical designs, frameworks combining the Cloud, Edge, and Terminal (MD) layers [29] present one of the closest architectural archetypes to the full four-tier continuum. Rather than proposing explicit algorithmic solutions, certain works provide essential theoretical foundations. The study in [31] offers a comprehensive survey comparing Cloud, Edge, and Fog information technologies on an application-dependent basis. While Ref. [32] compares the technical parameters of Fog, Cloudlet, and MEC to establish a decision tree for the initial framework design phase.

To scan the complex solution space, a broad methodological spectrum ranging from deterministic mathematical models to advanced meta-heuristic algorithms has been applied in the literature. Within the domain of mathematical programming, Semidefinite Relaxation (SDR)-based techniques have been utilized to minimize the weighted cost of energy consumption and delay under multi-user structures and resource (bandwidth, power) constraints in Mixed Fog/Cloud systems [26]. Similarly, SDR-based algorithms have been proposed for task offloading from a single mobile device to multiple end devices while jointly optimizing energy, latency, and CPU frequency scaling [33]. In addition to analytical models, game-theoretic approaches such as the Wardrop Equilibrium have been introduced to solve task offloading in Edge–Cloud networks by classifying tasks as hard or soft based on time constraints [25].

Regarding classic heuristics and meta-heuristics, three distinct CMOEAs were developed to simultaneously optimize energy consumption and time constraints [24], while a discrete version of the Non-dominated Sorting Genetic Algorithm II (D-NSGA-II) was implemented using classical evolutionary operators for delay minimization in Fog–Cloud systems [23]. Under the swarm intelligence paradigm, a Non-dominated PSO (NPSO) algorithm was introduced to achieve an energy–delay balance in multi-application scenarios such as smart agriculture [28]. Furthermore, control designs leveraging PSO have been explored for IoT applications using Edge–Cloud layers, though operating within Model Predictive Control (MPC) frameworks rather than explicit task offloading [34]. To address processing costs and load balancing in Cloud environments, an Enhanced Firefly Algorithm (IFA) was driven to optimize execution parameters [35]. Additionally, hybrid meta-heuristics have been formulated to balance the exploration and exploitation phases in the search process, specifically aiming to mitigate local solution stagnation and premature convergence issues [21].

To cope with the high volatility and uncertainties of dynamic IoT environments, learning-guided and artificially intelligent mechanisms have recently gained prominence. Within the scope of deep learning, a Deep Fusion Generative Adversarial Network (DFGAN) framework was introduced to simultaneously optimize multiple objectives including latency, cost, load balancing, and resource utilization across Cloud, Edge, and Terminal layers [29]. Similarly, a Distributed Deep Learning-based Offloading (DDLO) method was implemented to handle application-based latency constraints and maintain an energy–latency balance in multi-user multi-server MEC networks [30].

When online learning and intelligent exploration of unknown Edge nodes are required, Multi-Armed Bandit (MAB) learning approaches offer viable alternatives. In this context, a standard Upper Confidence Bound (UCB1) reinforcement learning approach was applied to jointly optimize energy consumption and latency in maritime IoT networks. The authors further introduced a novel UCB variant, namely UCB1-ESSS, to adaptively balance advantages and costs during the Edge server selection process [36]. Although these learning-driven models provide remarkable adaptability, existing solutions in the literature either rely on pure machine learning architectures without hybridizing with robust meta-heuristics such as ACO or PSO, or they remain restricted to highly specialized, domain-specific deployment areas like maritime or industrial scenarios.

### 2.3. Problem Complexity

In Cloud and Fog computing-based network architectures, energy efficiency and low latency are the most fundamental performance metrics determining the operational success of the system. The limited battery capacity of Edge devices (IoT nodes) makes minimizing energy consumption essential for extending the network’s lifespan. Furthermore, latency directly impacts the quality of service (QoS) of critical applications, especially those requiring real-time response, and determines the reliability of the system.

In task-offloading processes, energy consumption and latency typically appear as conflicting objectives, which makes it difficult to optimize both simultaneously. Although transferring a task from a local endpoint to a more powerful Edge, Fog, or Cloud node saves processing energy, it increases network latency and communication-related energy costs. On the other hand, aggressive computational choices aimed at minimizing latency can result in an excessive energy load on nodes with limited resources. This situation results in the problem taking on a multi-objective optimization structure and requires the system to find an acceptable trade-off point between the two metrics.

### 2.4. Optimization Framework

The Mathematical Nature of Task-Offloading Processes: Task offloading problems inherently have a discrete, global, and multi-objective optimization structure. The problem exhibits a discrete nature because the decision to assign tasks to a specific node or keep them locally requires a binary (“0–1”) integer-based selection process. Moreover, the fact that the optimal solution involves searching the entire search space in variable network topologies places the problem within the category of global optimization. The need to simultaneously optimize conflicting objectives such as energy efficiency, computational latency, and bandwidth usage further reinforces the multi-objective nature of the problem. When these complex characteristics are put together, the problem’s solution space grows exponentially with the number of tasks and nodes, and the solution process reaches an NP-hard level.

A review of the discrete, global and multi-objective nature of task offloading studies in the literature is presented below.

*Discreteness*: Many formulations use binary or mixed-integer variables to indicate whether a task (or subtask) is executed locally, at an Edge server, at a Fog server, in the Cloud, or at another device:

- Task offloading is modeled as a mixed-integer non-linear programming (MINLP) problem in multi-UAV MEC and solved via splitting and rounding methods [37].
- Cooperative MEC offloading is cast as a bi-level problem where upper-level offloading decisions are constructed by an ant colony system over discrete modes (local/cooperative/server) [38].
- Gray wolf-based WOLVERINE explicitly uses a binary multi-objective GWO algorithm for offloading decisions in collaborative MEC [39].
- Dependent-task offloading in MEC is formulated as an NP-hard problem with binary assignment of subtasks to heterogeneous nodes, solved via multi-objective PSO [40].
- Several multi-objective evolutionary or PSO approaches encode offloading as discrete genes/particles, sometimes with partial-offload ratios as additional continuous variables, leading to hybrid discrete–continuous spaces [39,41,42].

These models clearly implement discretization through 0–1 (or small integer) variables that specify the offload location or mode, and this is typically combined with continuous resource allocation decisions [37,38,41,43].

*Global Optimality*: Suboptimal trapping creates a risk during local search, because offloading spans heterogeneous tiers and nodes (e.g., end, Edge, Fog, Cloud, UAVs, satellites, vehicles).

- Multi-tier and collaborative settings, such as Cloud–Edge–end, MEC–vehicular Fog, and Terminal–satellite–Cloud, are modeled as NP-hard MINLP/CMOP problems. This makes meta-heuristics or DRL necessary for global exploration [37,43,44,45,46].
- WOLVERINE specifically uses the evolutionary, multi-objective GWO algorithm to explore the global Pareto front in highly coupled, NP-hard search space [39].
- Bi-level CoMECO offloading uses ACO with a local search to find approximate solutions to problems involving strong coupling between discrete offloading and continuous resources [38].
- Surveys and related work sections emphasize that greedy or heuristic local strategies can become trapped in local optima, particularly in dependent tasks and heterogeneous layers [40,45,47].

Offloading should be treated as a global (combinatorial) search rather than a local optimization, especially when it is used on a large scale.

*Multi-objectivity*: A significant body of research explicitly defines offloading as multi-objective optimization, typically balancing factors such as delay/latency, energy consumption, and occasionally monetary or resource cost:

- Delay-energy CMOP in MEC/Fog is solved via evolutionary algorithms to obtain a Pareto optimal front [43].
- Latency, energy usage, and resource costs are optimized jointly via a multi-objective framework using binary GWO offloading [39].
- Time delay, energy consumption, and task execution cost are minimized jointly by PSO in IIoT MEC [42].
- Multi-objective offloading algorithms in Cloud–Edge–end collaboration minimize average latency and energy consumption for complex, dependent tasks [47,48].
- Cloud–Edge collaborative schemes for 6G and vehicular/MEC networks minimize latency and energy by often aggregating them into system cost or exploring the full Pareto set using MOEAs or DRL [44,45,48,49,50].
- Earlier Fog–Cloud work minimizes a weighted combination of delay, energy, and Cloud payment. This is explicitly framed as a multi-objective problem and then relaxed [43].

These studies either (i) maintain a Pareto-based multi-objective view [39,40,43,45,47,48,50], or (ii) encode multi-objective trade-off using weighted sums or utility functions (e.g., latency–energy cost functions) [42,44,49,51]. As mentioned in both cases, the fundamental structure is multi-objective. Improving latency usually worsens energy or cost, and vice versa [39,43,47].

Three main approaches have emerged in the literature for solving complex network problems: exact methods, heuristic approaches, and meta-heuristic algorithms. While exact methods such as “Branch and Bound” or “Dynamic Programming” guarantee an exact optimal solution for small-scale networks, they are inadequate for large and hierarchical network scenarios due to computational costs. Methods such as Deep Reinforcement Learning (DRL), Game Theory, and meta-heuristics are widely used to overcome this bottleneck and facilitate real-time decision-making processes. These methods aim to generate “good enough” solutions that are as close as possible to the optimal result in a reasonable amount of time. They do this by scanning complex solution spaces with intelligent scanning mechanisms.

The main reason to prefer meta-heuristic, genetic, and bio-inspired optimization methods is that these algorithms exhibit a high balance of exploration and exploitation in complex, multi-dimensional search spaces. Methods such as ACO or GA demonstrate a high degree of resilience in reaching the global optimum without getting stuck at local optima by imitating collective intelligence found in nature. Especially in energy-constrained and dynamic IoT environments, the flexibility offered by these algorithms enables them to generate stable solutions under non-linear constraints without requiring the mathematical model derivative. These features make meta-heuristic approaches superior and more adaptive than traditional methods in the management of heterogeneous resources within hierarchical Edge–Fog–Cloud architectures.

#### Optimization Methods Used

In hierarchical Edge–Fog–Cloud architectures, the task-offloading decision requires a complex optimization process due to the size of the solution space and the NP-hard nature of the problem. The inefficiency of traditional mathematical modeling methods when applied to a large scale in terms of computational cost and dynamic networks have made meta-heuristic and bio-inspired algorithms widespread in the literature. In this subsection, an evaluation is conducted on how meta-heuristic algorithms—which stand out particularly for their global search capabilities and resistance to getting stuck in local optima—are adapted to task-offloading problems, the challenges encountered, and the proposed solutions in the literature.

To map out current trends and highlight essential factors in the field of offloading, review studies on offloading conducted over the past five years were examined.

This review states that, in the 35 studies examined, the most commonly used techniques were heuristic methods, suggesting a trend towards these methods in data offloading approaches [16]. Genetic algorithms are used to discover and develop the most suitable solutions for task scheduling, load balancing and resource allocation, taking various parameters and constraints into account in the field of Edge artificial intelligence and data offloading scenarios [12,15,18]. It has been emphasized that the greatest advantage of heuristics is their ‘acceptable cost’, but that they do not guarantee optimality [10,12]. The study states that, in optimization problems, a heuristic method is a technique that aims to solve the problem more quickly than traditional methods by compromising on completeness, accuracy, optimality or precision [14]. Heuristic algorithms rank first at 31.5% among Resource Allocation Techniques [10].

As heuristic algorithms are experience-based techniques that do not guarantee an optimal result, meta-heuristics provide general computational intelligence frameworks that can be adapted to different optimization problems with minimal modification and potentially offer a sufficiently good solution [14]. Particularly in task-scheduling processes within artificial intelligence-enabled Internet of Things (AIoT) systems, meta-heuristic methods such as GA and PSO offer critical efficiency in generating near-optimal solutions with low computational load for energy-constrained Edge devices [12]. In multi-objective optimization problems, a suitable Pareto front provides greater flexibility in selecting the solution that offers the best trade-off between optimization criteria. The most common solution to the problem of generating a Pareto set, which involves high computational costs, is through the use of meta-heuristics capable of obtaining a near-optimal front [6]. For these reasons, Meta-heuristic algorithms, which aim to find the optimal or near-optimal solutions to resource allocation problems in a reasonable timeframe, are used in 9.2% of cases [10]. On the other hand this study indicates that meta-heuristic methods can be used for large-scale optimization but highlights the difficulty of tuning sensitive hyperparameters [11].

Some research into task-offloading optimization has been conducted using heuristic and meta-heuristic methods; however, these methods have certain shortcomings. The main problem with meta-heuristic methods is that they can become trapped in local optima. To obtain a globally optimal solution, they must strike a balance between exploration and exploitation. Furthermore, meta-heuristic algorithms have numerous hyperparameters, which are often difficult to tune. These are parameters that must be set manually by the researcher [13].

Although different objectives need to be optimized simultaneously, they are often inconsistent or incompatible, frequently requiring different trade-offs and compromises. This leads researchers to frequently use a mixture of various heuristic algorithms to address critical parameters in resource allocation and workflow planning [7].

The modern literature addresses task-offloading optimization in MEC and IoT networks under three main categories: classical, heuristic, and artificial intelligence-based methods. Whilst classical methods guarantee a global optimum, the exponential growth of the search space in complex network structures renders these approaches unsuitable for large-scale systems. Although heuristic and meta-heuristic methods offer ‘near-optimal’ solutions by increasing convergence speed, the need to reconfigure the algorithm in scenarios where environmental parameters change dynamically poses a significant constraint. At this point, the development of robust approaches that gradually improve system performance through feedback mechanisms and offer higher adaptability constitutes the focus of current research [17].

Consequently, there is an increasing number of hybrid algorithms in the literature. Among the algorithms categorized as heuristic, meta-heuristic, and hybrid meta-heuristic algorithms, nearly half of the solutions fall into the heuristic category, whilst 39% of the studies are based on meta-heuristic algorithms. The remaining 12% are hybrid meta-heuristic algorithms [8]. The findings of this study suggest that hybrid meta-heuristic algorithms and the integration of machine learning could represent promising opportunities for future research [19].

In the conceptual hierarchy, standard heuristics are ‘shortcut’ rules developed specifically for a particular problem, which are fast but generally yield suboptimal results. Meta-heuristic frameworks, on the other hand, are general-purpose strategy templates that go beyond such simple rules and can be adapted to almost any type of optimization problem (such as ACO, PSO or Simulated Annealing). GAs are a very specific and powerful type within this meta-heuristic family, inspired by biological evolution. In summary: heuristics are problem-specific tactics, meta-heuristics are general game plans that govern these tactics, and GAs are one of the most popular versions of this plan that operates via evolutionary mechanisms. Meta-heuristic frameworks demonstrate greater resilience compared to standard heuristic methods by striking an effective balance between global exploration (diversification) and local exploitation (convergence); this typically leads to consistent and reliable performance across various optimization tasks [52].

**Figure 1:**
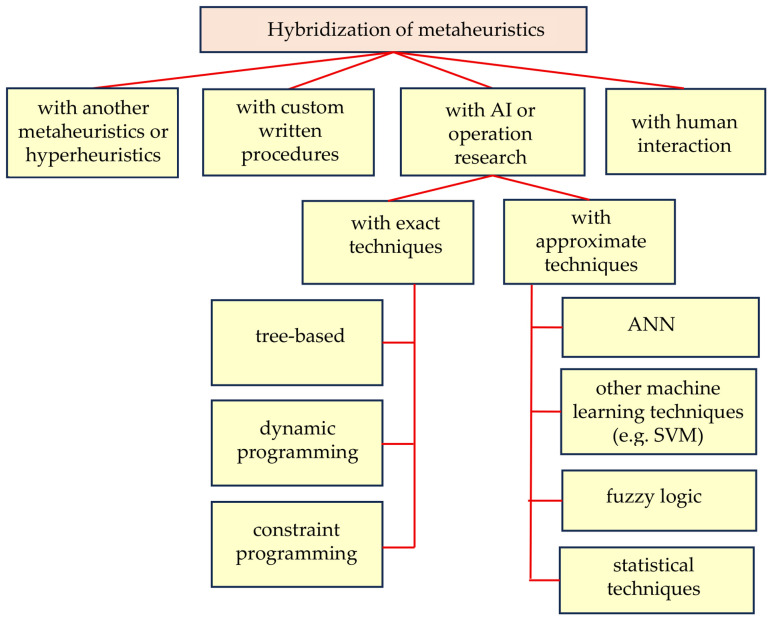
Meta-heuristic hybridization method classification. Adapted from [53].

There are numerous machine learning techniques, and many of these can be integrated into meta-heuristic frameworks to improve or guide the exploration of the solution space. Many machine learning techniques can enhance and enrich meta-heuristic methods. Machine learning can be used for training to predict promising regions of the search space, thereby allowing meta-heuristic methods to avoid a large portion of the exploration process and instead focus on regions where the best solutions are already expected. Convergence speed can thus be improved [53]. The topic of hybrid meta-heuristic methods is a highly comprehensive one. Comprehensive information on the methods, their taxonomy and approaches are provided in [52]. Figure 1 shows the classification of meta-heuristic hybridization methods, modified by [53] with reference to [52].

**Figure 2:**
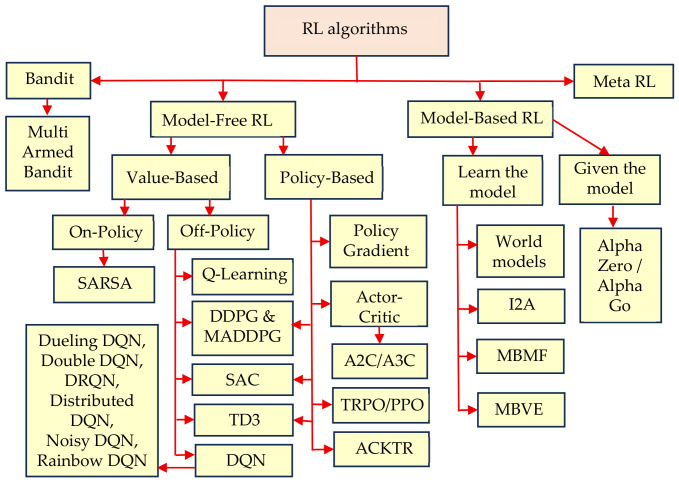
The taxonomy of RL algorithms. Adapted with permission from [13].

Reinforcement learning (RL) methods are frequently used in meta-heuristic hybridization. A taxonomy of RL algorithms is shown in Figure 2 [13]. The Multi-Armed Bandit (MAB) algorithm is also among the most important RL methods used in offloading [11,14]. Most RL policies face the challenge of striking a balance between exploration and exploitation. There must be a balance between searching for the problem space for profitable actions and executing the best possible trial action. The MAB problem is one of the earliest examples of the exploration–exploitation trade-off. It aims to strike a trade-off between following the best past solution and discovering potential new opportunities. At each step, a single resource is allocated to an action in an MAB problem, yielding an observable reward. The objective is to maximize the total profit gained across a series of allocations. The agent must learn to select a single optimal branch from a set of candidate branches based on previous rewards [13].

The results of the literature review also indicate that combining the ‘restart cost’ disadvantage of heuristics (such as ACO) with UCB’s ‘learning from feedback’ capability constitutes a consistent and robust solution.

### 2.5. Research Gap and Positioning of the Proposed Study

The literature review began by examining systematic review articles from the past five years to provide an up-to-date overview of the current state of the field. This preliminary phase aims to identify the general trends and evolution of task-offloading strategies in heterogeneous environments. Research on energy-efficiency and latency-aware optimization models, providing a comprehensive analysis of studies that reflect the discrete, global, and multi-objective nature of these complex problems. Despite the abundance of existing research, there remains a significant gap in the literature regarding the dynamic management of the exploration and exploitation balance under context-sensitive constraints. To clarify how the proposed study addresses this gap and situates itself within the literature, a comparison table outlining the key differences between the proposed study and current methodologies is provided in Table 1.

In conclusion, although the existing literature provides robust solutions for task offloading, a critical balance problem remains between the sensitivity of pheromone-based exploitation and the necessity of uncertainty-aware exploration in highly dynamic environments. Most studies either rely on static heuristic weights or fail to adapt to the multi-objective nature of diverse application profiles. The proposed UCB-ACO framework addresses this fundamental gap by integrating the UCB strategy within the ACO framework, ensuring a context-aware and balanced decision-making process.

After establishing the conceptual framework and necessity of this hybrid approach, the next section presents the Problem Formulation, which includes the mathematical network model and multi-objective cost functions that manage the optimization process.

## 3. Problem Formulation

This section outlines the formal structure of the proposed task-offloading framework, which is based on the complex interaction between distributed computing resources and various user requirements. Here, the framework considered has been modeled as a multi-dimensional ecosystem that combines multi-server, multi-task, multi-user, and multi-application capabilities within a hierarchical structure. The key difference that sets the proposed work apart from existing approaches in the literature is that it implements a comprehensive four-tier architecture that utilizes context-aware optimization to simultaneously balance latency and energy.

To establish a solid foundation for the UCB-ACO algorithm, the problem has been divided into two main components. First, Section 3.1 details the physical and logical structure of the hierarchical system. Next, Section 3.2 presents the mathematical definitions and multi-objective cost functions that govern the decision-making process.

### 3.1. Hierarchical Four-Tier System Model

**Figure 3:**
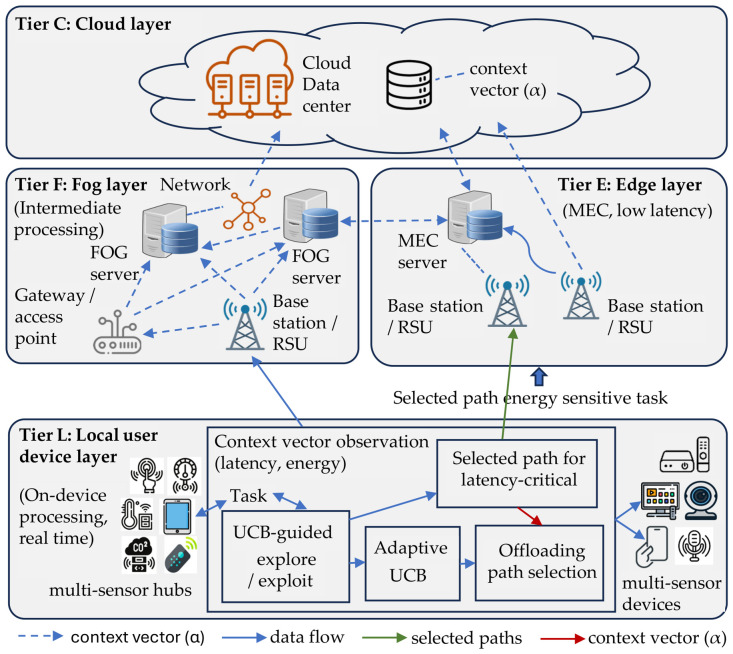
The proposed hierarchical four-tier system model.

As shown in Figure 3, the proposed hierarchical four-tier system model represents a heterogeneous environment characterized by its multi-server, multi-task, multi-user, and multi-application architecture.

The architecture is structured into four fundamental tiers to address diverse computing requirements: the local device tier (mobile terminals), the Edge tier (base stations and access points), the Fog tier (local micro data centers), and the Cloud tier (central data centers). In this study, to clearly evaluate the efficiency of the proposed UCB-ACO algorithm, it is assumed that all nodes (mobile devices) are stationary. This assumption allows the model to focus primarily on the resource allocation and offloading decision-making processes across the multi-Edge and multi-Fog nodes without the interference of mobility-sourced offloading latencies.

The integration of a dedicated Fog layer along with the Edge layer is a strategic requirement for managing high-density, heterogeneous task traffic. Here, this distinction is supported by the literature indicating that “Fog computing has a hierarchical nature, while Edge computing is limited only to a few end devices. Fog computing can also address data storage, data control and data processing acceleration while Edge computing is not capable of these services [54]”. By utilizing this hierarchical depth, the system can effectively classify and distribute tasks according to their specific latency-critical or energy-sensitive profiles. Mathematically, this dedicated intermediate layer prevents resource over-allocation at the local Edge access points while shielding the core Cloud backhaul from bandwidth saturation. Consequently, the dedicated Fog tier expands the feasible solution space for the proposed learning-guided meta-heuristic, enabling optimal multi-objective trade-offs between execution delay and local energy depletion. The following section presents a detailed mathematical model and objective functions to quantify the interactions within this complex hierarchy and identify the constraints of the optimization process.

### 3.2. Mathematical Model

This section details the mathematical foundations and optimization objectives of the proposed four-tier hierarchical task-offloading architecture. The model’s structure begins with a presentation of the basic indices and set definitions used in the system. After it explains the decision mechanism and objective functions built upon these foundations. The proposed model is based on a multi-objective and multi-user architecture designed to maximize the system’s success rate and reliability while minimizing latency and energy consumption. The system’s decision-making mechanism, computational costs, and constraint management are detailed below.

#### 3.2.1. Indices and Sets

The basic indices, sets, and their value ranges used to ensure the consistency of the mathematical model are defined below:

- i∈{1,2,…,N}: Index of mobile users and their corresponding tasks. It is defined as an integer vector ranging from 10 to 100 according to simulation scenario N.
- j∈{L,E,F,C}: Set of hierarchical tiers representing local (L), Edge (E), Fog (F), and Cloud (C).
- k∈{1,2,3}: Index of optimization objectives (latency, energy, cost).

#### 3.2.2. Decision Variables

The fundamental decision-making process in the system is based on assigning tasks to layers and evaluating these assignments within the constraints of the physical network.

The fundamental decision is represented by a binary variable $x_{i,j}$, which indicates the offloading destination:

(1) $$x_{i,j}=\left\{\begin{array}{ll} 1, & \mathrm{if}\;\mathrm{task}\;i\;\mathrm{is}\;\mathrm{offloaded}\;\mathrm{to}\;\mathrm{tier}\;j\\ 0, & \mathrm{otherwise} \end{array}\right.$$

Constraint: Each task must be assigned to exactly one tier:

(2) $$\sum_{j\in \{L,E,F,C\}}x_{i,j}=1,\;\forall i\in \left\{1,\ldots ,N\right\}$$

The structural integrity of the task allocation matrix is governed by the constraint in Equation (2). Since $x_{i,j}$ is strictly a binary variable restricted to $\left\{0,1\right\}$, enforcing the summation over the tier domain $\left\{L,E,F,C\right\}$ to equal exactly unity mathematically prevents two invalid operational states: under-assignment (where a task is dropped, resulting in a sum of 0) and over-assignment (where a task is duplicated across multiple tiers, resulting in a sum ≥2). In the hybrid UCB-ACO algorithm, this mathematical boundary is natively integrated. Therefore, during path construction, each heuristic particle only chooses one discrete tier destination for each task, making sure all generated solution vectors are structurally feasible.

Data Transfer Rate (Wireless Data Rate): The bit-per-second transmission capacity ($R_{i,j}$) across the heterogenous tiers is governed by the underlying physical layer channel state and interference metrics. To maintain a strict focus on the proposed multi-tier optimization framework and algorithmic decision space, the foundational physical layer equations derived from the Shannon–Hartley theorem [55,56]—including the comprehensive formulation of $R_{i,j}$ and the Signal-to-Interference-to-Noise Ratio (SINR)—are delegated to Appendix A. The resulting transmission rate $R_{i,j}$ directly parameterizes the tier-specific communication latencies as formulated below.

Layer-Based Transmission Delays (Comm. Delays) are the time elapsed for data to travel from the device to the relevant layer server.

Local Transmission Delay (the transmission delay at the local device (j=1)) is considered to be zero.

(3) $$T_{i,1}^{comm}=0$$

Edge Transmission Latency is the time for data to be transmitted to the Edge server.

(4) $$T_{i,2}^{comm}=\frac{D_{i}}{R_{i,2}}$$

Fog Transmission Delay is the time for data to be transmitted to the Fog layer.

(5) $$T_{i,3}^{comm}=\frac{D_{i}}{R_{i,3}}$$

Cloud Transmission Latency is the time it takes for data to be transmitted to the Cloud server.

(6) $$T_{i,4}^{comm}=\frac{D_{i}}{R_{i,4}}$$

Delay in general is defined as

(7) $$T_{i,j}^{comm}=\frac{D_{i}}{R_{i,j}}\;,\;\;\;\;\;\;\;j\;\in \{2,3,4\}$$

Total Communication Delay is the total transmission time resulting from the selected layer [57,58]:

(8) $$T_{i}^{comm}=\sum_{j=1}^{4}x_{i,j}T_{i,j}^{comm}.$$

Device Transmission Energy is the energy consumed by the device when sending task *i* to the server at layer *j* over the wireless channel:

(9) $$E_{i,j}^{trans}=P_{i}\cdot T_{i,j}^{comm}.$$

Download Energy is the energy consumed during the computation result retrieval:

(10) $$E_{i,j}^{rec}=P_{rec}\cdot T_{i,j}^{down}.$$

Total Communication Energy is the total energy consumed for task transfer:

(11) $$E_{i}^{comm}=E_{i,j}^{trans}+E_{i,j}^{rec}.$$

#### 3.2.3. Mathematical Modeling of Objectives

This section defines cost analyses for the computation phase of tasks and performance metrics that measure the system’s overall performance. Following the communication phase (Section 3.2.2), the computation times for each task on the assigned layer and the amount of energy consumed by the mobile device during this process are calculated. In the remainder of this section, the criteria of delay, energy efficiency, success rate, and reliability—which characterize the proposed model’s versatile structure—are mathematically defined. These models serve as the foundation for validating the performance metrics (such as success rate and reliability) presented in the simulation results.

##### Computational and Energy Model

Local Execution Time: The time it takes for the task to complete on the mobile device’s own processor ($f_{L}$).

(12) $$T_{i,1}^{comp}=C_{i}/f_{L}$$

Edge Processing Time: The duration calculated based on the capacity of the Edge server ($f_{E}$).

(13) $$T_{i,2}^{comp}=C_{i}/f_{E}$$

Fog Processing Time: The duration calculated based on the Fog server’s capacity ($f_{F}$).

(14) $$T_{i,3}^{comp}=C_{i}/f_{F}$$

Cloud Processing Time: The duration calculated based on the capacity of the Cloud server ($f_{C}$).

(15) $$T_{i,4}^{comp}=C_{i}/f_{C}$$

Overall processing time is required to complete the task, depending on the hardware capacity of the relevant layer [24,59].

(16) $$T_{i,j}^{comp}=\frac{C_{i}}{f_{j}}\;\;\;\forall j\;\in \{1,2,3,4\}$$

Local Energy Consumption: The energy consumed by the device while using its own processor.

(17) $$E_{i,L}^{comp}=\kappa \cdot C_{i}\cdot f_{L}^{2}$$

Remote Processing Energy: The processor power consumption of a mobile device when tasks are offloaded to the outer layers (Edge, Fog, or Cloud layers). For tasks processed remotely, the device’s processing power is negligible.

(18) $$E_{i,remote}^{comp}\approx 0,\;\;\;\;j\;\in \{2,3,4\}$$

Total Latency: The sum of transmission, processing, and queueing times.

(19) $$T_{total,i}=T_{i}^{comm}+T_{i}^{comp}+T_{i}^{wait}+T_{i}^{overhead}$$

Here, $T_{i}^{overhead}$ accounts for the deterministic system and control-plane overheads, including network connection setup (e.g., protocol handshakes), task routing, and scheduling delays. In accordance with the standard Edge–Fog orchestration literature [59,60,61], this parameter is modeled as a constant 5 ms (0.005 s) per task in the simulation environment, while dynamic queueing wait times are abstracted away ($T_{i}^{wait}=0$) to maintain computational efficiency across extensive meta-heuristic evaluations.

Total Energy: The sum of communication and local processing energy [24,62].

(20) $$E_{total,i}=E_{i}^{comm}+E_{i}^{comp}$$

##### Multi-Objective Optimization and Metrics

Objective 1—Delay Minimization $\left(f_{1}\right)$: The system’s primary time performance objective.

(21) $$minf_{1}=\sum_{i=1}^{N}T_{total,i}$$

Objective 2—Energy Minimization $\left(f_{2}\right)$: The battery-preserving objective of the device.

(22) $$minf_{2}=\sum_{i=1}^{N}E_{total,i}$$

Task Success Condition ($\psi_{i}$): The condition under which the task meets both the time and energy constraints.

(23) $$\psi_{i}=\left\{\begin{array}{ll} 1, & T_{i}\leq T_{\max,i}\;\wedge \;E_{i}\leq E_{\max,i}\\ 0, & \mathrm{otherwise} \end{array}\right.$$

Success Rate (SR): The overall efficiency rate of a system used in comparing algorithms.

(24) $$SR=\left(\frac{1}{N}\sum_{i=1}^{N}\psi_{i}\right)\times 100\%$$

Objective 3—Reliability Maximizing ($f_{3}$): This leads to having an ability to avoid constraint violations.

(25) $$maxf_{3}=Reliability=1-CVR$$

(26) $$maxf_{3}=1-\frac{1}{N}\sum_{i=1}^{N}I(Violation)$$

#### 3.2.4. Context-Aware Aggregate Objective Function (J)

This section explains how sub-objectives (latency, energy consumption, and reliability), each with different physical units and priorities, are combined under a single scalar cost function. The proposed model uses a weighted sum function that can dynamically adapt to the varying needs of application profiles. These application profiles can be real-time data processing or energy-efficient sensor networks. In addition, to ensure the physical limits of the system (deadline and battery capacity) are maintained, a dynamic mechanism has been designed. This mechanism incorporates constraint violations into the cost function as a penalty term. This approach ensures that the optimization algorithm converges not only to low-cost solutions but also to feasible ones.

Combined Fitness Function/Cost Function (J) The aim is to solve this multi-objective problem effectively without losing constraint stringency. So, the primary execution performance indices and the structural reliability parameters are dynamically blended into a unified scalarized fitness function (Fitness) representing the global optimization landscape:

(27) $$Fitness={w_{1}f_{1}}^{\prime }+{w_{2}f_{2}}^{\prime }+\left(1-f_{3}\right)$$

where ${f_{1}}^{\prime }$ and ${f_{2}}^{\prime }$ represent the normalized variants of delay and energy minimization objectives, respectively, intended to eliminate scaling dimensional differences. The relative importance of these physical resource indices is governed by the application profile weights, satisfying the strict convex constraint:

(28) $$w_{1}+w_{2}=1,\;w_{k}\geq 0\;$$

The goal is to eliminate dimensional scaling differences and ensure objective parity. Hence a linear Min-Max normalization technique is deployed to map both raw execution delay ($f_{1}$) and raw energy consumption ($f_{2}$) values into a uniform dimensionless range of $\left[0,1\right]$. The normalized objectives ${f_{1}}^{\prime }$ and ${f_{2}}^{\prime }$ are formally calculated as follows:

(29) $${f_{1}}^{\prime }=\frac{f_{1}-f_{1}^{min}}{f_{1}^{max}-f_{1}^{min}}$$

(30) $${f_{2}}^{\prime }=\frac{f_{2}-f_{2}^{min}}{f_{2}^{max}-f_{2}^{min}}$$

where $f_{k}^{min}$ and $f_{k}^{max}$ ($k\in \left\{1,2\right\}$) denote the minimum and maximum operational boundaries, respectively, observed for delay and energy within the multi-tier network simulation environment. For the reliability score ($f_{3}$), since it is inherently defined as a probability metric spanning $\left[0,1\right]$, it is directly incorporated into the cost function via its complement $\left(1-f_{3}\right)$ to align with the minimization trajectory of the hybrid framework.

To mitigate execution delays and mobile device energy depletion under strict reliability constraints, this global task-offloading problem is multi-objective and non-linear. The mathematical framework developed from Equation (21) to Equation (27) establishes a rigid optimization space. It is necessary to bridge the physical gap between distinct metrics operating in different dimensions (seconds versus Joules). So, the primary objective functions *f*_1_ (delay minimization) and *f*_2_ (energy minimization) are mapped onto a single scalar cost landscape via dynamic normalization, represented as *f*_1_′ and *f*_2_′ in Equation (27).

Crucially, the system optimization landscape preserves its strict tri-objective dimensionality despite utilizing a bi-objective preference weight vector. In this architecture, physical resource metrics ($f_{1}$ and $f_{2}$) represent elastic domains where dynamic compromises are established depending on the deployment profile. Conversely, system reliability ($f_{3}$) represents an inelastic survivability criterion that cannot be degraded or softened by arbitrary weights. By embedding the Constraint Violation Rate (CVR) via the unweighted term $\left(1-f_{3}\right)$ directly into Equation (27), reliability acts as a global architectural anchor.

In the resource-constrained operation mode (Case B), any violation of the deterministic task deadlines (Equation (31)) or battery capacities (Equation (32)) triggers the external hybrid step-linear penalty framework $P\left(x\right)$ defined in Equations (35) and (36). This penalty artificially inflates the global cost of invalid tracks, forcing the hybrid UCB-ACO learning particles to reject unstable states and converge exclusively onto robust, Pareto-optimal offloading trajectories where $\left(1-f_{3}\right)\to 0$.

The latency constraint specifies the maximum time limit allowed for completing each task.

(31) $$T_{total,i}\leq T_{max,i}\;\;\;\;\;\forall i\in \left\{1,\ldots ,N\right\}$$

The energy constraint is the maximum amount of energy a mobile device can allocate to a single task. It limits the energy cost of each task assignment to preserve the device’s battery life.

(32) $$E_{total,i}\leq E_{max,i}\;\;\;\;\forall i\in \left\{1,\ldots ,N\right\}$$

Constraint Violation Rate (CVR) is the rate of out-of-rule assignments among all tasks. It is a statistical metric that measures how well the system follows constraints.

(33) $$CVR=\frac{1}{N}\sum I\left(\mathrm{Constraint}\;\mathrm{Violation}\right)$$

(34) $$CVR=\frac{1}{N}\sum I\left(T_{total,i}>T_{max,i}\;\mathrm{or}\;E_{total,i}>E_{max,i}\right)$$

By using the indicator function inside the Constraint Violation Rate (CVR), any violation of the deterministic boundaries defined in Equations (31) and (32) is strictly penalized using the two-tier step-linear formulation of the penalty term in Equation (36). The objective function effectively transforms into an adaptive landscape where unfeasible task assignments are heavily penalized, forcing the meta-heuristic ants and learning particles to converge only on Pareto-optimal solutions.

The penalty function is a mathematical deterrent that directly influences the results of Case B (Constrained Case). By artificially increasing the fitness value of solutions that violate the constraints (thereby increasing their cost), it ensures that the algorithm avoids these regions [63,64].

(35a) $$P_{T}\left(x_{i}\right)=\left\{\begin{array}{cc} 0, & \mathrm{if}\;T_{total,i}\leq T_{max,i}\\ \alpha +\beta \cdot \left(T_{total,i}-T_{max,i}\right), & \mathrm{if}\;T_{total,i}>T_{max,i} \end{array}\right.$$

(35b) $$P_{E}\left(x_{i}\right)=\left\{\begin{array}{cc} 0, & \mathrm{if}\;E_{total,i}\leq E_{rem,i}\\ \alpha +\beta \cdot \left(E_{total,i}-E_{rem,i}\right), & \mathrm{if}\;E_{total,i}>E_{rem,i} \end{array}\right.$$

(36) $$P\left(x\right)=\sum_{i=1}^{N}\left[P_{T}\left(x_{i}\right)+P_{E}\left(x_{i}\right)\right]$$

In the proposed model, the current energy status (E_rem_) for each device is monitored, and the total energy consumption (E_total_) is calculated solely based on the hardware components (antenna and CPU) of the mobile device. A high-cost penalty function constrains the solution space if the remaining energy is insufficient.

It is aimed to effectively enforce the operational constraints defined in Case B and to ensure the reproducibility of the evaluation. For this reason, the proposed architecture employs a Hybrid Step-Linear Penalty Scaling mechanism. Unlike traditional quadratic scaling, this formulation applies a two-tier penalization strategy. When a solution violates strict architectural boundaries (i.e., exceeding the maximum task deadline or the remaining energy capacity), the penalty is applied.

Specifically, if a constraint is breached, the fitness function immediately imposes a heavy static baseline penalty (α=1000). This step-function deterrent instantly degrades the fitness of unfeasible assignments, strictly segregating them from feasible solutions. Furthermore, to differentiate between minor and severe violations, a dynamic linear penalty factor (β=500) is multiplied by the exact magnitude of the constraint breach and added to the baseline penalty.

This hybrid structure is mathematically strategic. The massive static penalty guarantees that constraint violations are heavily deterred, while the dynamic linear component creates a discernible fitness gradient. This gradient provides crucial directional information to the meta-heuristic search agents. It allows them to evaluate which unfeasible solutions are closer to the threshold and progressively guide them back into the feasible region without losing search diversity.

#### 3.2.5. Application Profiles and Dynamic Weighting

Adaptability to distinct application requirements and localized efficiency gains are discussed in this section. Edge/Cloud applications can be effectively clustered into specific groups based on their execution priorities despite the large variety of network traffic [65].

In the proposed framework, these varying operational requirements are dynamically handled through the weight coefficients $w_{1,app}$ and $w_{2,app}$. Thus, resource-allocation solutions tailored to specific application profiles can be generated.

The optimization objectives representing localized user preferences focus strictly on the independent physical domains of execution delay ($T_{i,j}$) and network energy depletion ($E_{i,j}$). This ensures absolute mathematical clarity and eliminates nomenclature redundancy. Accordingly, the local optimization goal is formulated to minimize the weighted sum of these performance indices normalized across the multi-tier system:

(37) $$minJ=\sum_{i=1}^{N}\sum_{j\in \{L,E,F,C\}}\left(w_{1,app}\cdot T_{i,j}+w_{2,app}\cdot E_{i,j}\right)\;\;\;\;\;\;\;\;\;\;\;$$

Subject to strict bi-objective convex weight constraints:

(38) $$\sum_{k=1}^{2}w_{k,app}=1,\;w_{k,app}\geq 0$$

The weight configuration vector $\left(w_{1,app},w_{2,app}\right)$ designated for each defined application profile during the simulation setup is explicitly detailed in Table 2.

It is worth noting that while the application-specific tuning weights are formulated as a bi-objective trade-off $(w_{1,app}+w_{2,app}=1$), the core computational framework preserves its tri-objective dimensionality. In multi-objective network designs, physical resource indices (execution time vs. power depletion) represent elastic domains where elastic compromises can be established based on the application profile. Conversely, system reliability ($f_{3}$) and constraint validation bounds represent inelastic system survivability criteria. Rather than deflating the mathematical rigor by assigning arbitrary soft weights to reliability across different apps, the proposed framework handles $f_{3}$ and the quadratic penalty $P\left(x\right)$ as a rigid baseline optimization vector. Consequently, the optimization engine continuously maps solutions onto a three-dimensional Pareto landscape, ensuring that user-centric performance adaptations never compromise structural network survivability.

This section has defined the formal mathematical constraints and cost functions characterizing the multi-tier task-offloading problem. The primary challenge here is to find the optimal $x_{i,j}$ assignment that minimizes the joint objective function J in a large-scale, heterogeneous search space. To solve this discrete and non-linear optimization problem, the architectural design and operational steps of the proposed hybrid UCB-ACO algorithm will be detailed in the next section.

## 4. Methodology

In this section, the hybrid UCB-ACO algorithm- one of the main contributions of this study—is described in detail, explaining how the UCB mechanism overcomes the stability limitations of standard ACO under stochastic channel conditions. In this context, a Hybrid Learning-Optimization framework has been adopted to overcome the limitations of pure biomimetic algorithms in dynamic IoT environments. Inspired by the highly effective exploration–exploitation balancing mechanism of the MAB problem, the proposed method establishes a ‘Learning-guided Meta-heuristic’ framework. Rather than deploying a formal statistical reinforcement learning policy, this framework integrates ACO with a UCB-inspired empirical learning mechanism. In this architecture, the ‘learning-guided’ aspect signifies that the meta-heuristic search continuously learns from the historical reward trajectories of previous iterations via the UCB index. This allows the algorithmic agents to adaptively adjust their search directions, dynamically balancing exploration and exploitation without relying on the strict statistical assumptions required by pure MAB theory. Finally, to ensure the scalability of the study, the simulation environment, tier-specific configurations, and simulation parameters were defined.

### 4.1. Hybrid UCB-ACO Algorithm Design

Traditional ACO yields successful results in complex search spaces. But it faces two main challenges, particularly in high-dimensional and dynamic Edge–Cloud systems: early convergence and getting stuck in local minima. In the standard ACO mechanism, ants tend to prefer paths with high pheromone density. This leads to the neglect of paths that have not yet been discovered but may be more optimal. The result is causing the algorithm to become trapped in its own self-cycle and delaying the discovery of the global optimum.

To overcome this structural limitation, this study introduces a “Learning-guided Meta-heuristic Approach” by embedding a UCB-inspired adaptation mechanism into the meta-heuristic framework. As a dynamic exploration bonus, the system uses the UCB index design rather than a rigid statistical RL agent. Using this hybrid architecture, the optimization process tracks not only the historical performance (exploitation) of each task-offloading track but also how infrequently a specific computing tier has been evaluated (exploration).

This approach is characterized as a “learning-guided” meta-heuristic, because the pheromone-driven path selection is continuously reinforced by an empirical feedback loop. In each iteration, the UCB index dynamically estimates the potential value of each computing tier (Edge, Fog, Cloud). This conceptual “optimism in the face of uncertainty” allows the ants to adapt to fluctuating network workloads and volatile channel conditions. By incorporating this dynamic uncertainty-driven bonus, the framework prevents premature pheromone saturation, thus successfully mitigating the slow-convergence bottleneck of traditional ACO and accelerating the global search trajectory.

The mathematical framework of the proposed hybrid UCB-ACO algorithm is formulated to refine the ants’ decision-making processes through this adaptive mechanism. The set of equations from (37) to (42) details the integration of the UCB index into the probabilistic transition rules and the subsequent pheromone updates. This formulation establishes an algorithmic mechanism. And elucidates how the system actively escapes local minima and balances exploration–exploitation in dynamic environments.

#### Mathematical Formalization of the Task Context Vector

Each task arrival is modeled alongside a multi-dimensional context vector ($X_{i}$). In doing so the learning-guided adaptive decision-making process in dynamic environments is supported. The context vector encapsulates the specific workload profile and resource constraints of the incoming task. Hence it serves as the foundational state knowledge that prevents the reinforcement learning agent from operating blindly. For each task i generated in the multi-tier simulation environment, the context vector is mathematically defined as follows:

(39) $$X_{i}=\left[D_{i},C_{i},\tau_{i}\right]$$

where $D_{i}$ denotes the instantaneous data size of the task (safeguarded within the realistic bounds of $\left[10,100\right]$ MB), $C_{i}$ represents the required computational cycles for CPU processing (bounded between $\left[1.0,5.0\right]$ Megacycles), and $\tau_{i}$ enforces the strict delay deadline constraint (ranging from $\left[0.1,0.5\right]$ s). At each execution step, the hybrid UCB-ACO framework observes this state vector $X_{i}$ directly from the task profile matrix. To calculate the physical limits and penalty parameters, the selection strategy uses these dynamic dimensions. In that way ensuring that the exploration–exploitation trajectory is tightly coupled with real-time application demands rather than static historical probabilities.

The UCB Selection Index serves as the operational criterion for balancing discovery and optimization. The index $U_{i,j}$ for mapping a task to a target server node is defined as follows:

(40) $$UCB_{i,j}\left(t\right)={\bar{\mu }}_{i,j}\left(t\right)+c\sqrt{\frac{\ln t}{n_{i,j}\left(t\right)}}$$

where ${\bar{\mu }}_{i,j}$ represents the empirical mean success (based on latency and energy metrics) accumulated by selecting node j up to the current iteration, $n_{i,j}\left(t\right)$ denotes the historical allocation count of this specific configuration, and c is a tunable exploration–exploitation parameter.

The mathematical formulation of the square-root uncertainty term in Equation (40) is conceptually rooted in Hoeffding’s Inequality [66], which historically provides probability bounds for tail distributions in MAB paradigms. The nature of real-world Edge–Cloud networks is non-i.i.d. (independent and identically distributed)—where queuing delays and stochastic channel states are inherently coupled—and highly dynamic. To accommodate this nature, this study adopts the UCB formulation not as a strict statistical bounding agent but as a heuristic learning guide. By utilizing the temporal horizon t and sample counts $n_{i,j}\left(t\right)$, the index establishes an adaptive confidence interval that mathematically forces the meta-heuristic search to diversify when uncertainty is high, effectively avoiding the premature convergence traps common in standard pheromone-driven networks.

Pheromone level ($\tau_{i,j}$) represents the preference intensity of paths in the ACO algorithm:

(41) $$\tau_{i,j}\left(t\right).$$

Hybrid Decision Matrix (Learning-guided Selection) is a combination of learning information from UCB and pheromone (τ) from ACO:

(42) $$Choice_{i,j}=\arg\max\left(U_{i,j}\left(t\right)\cdot \tau_{i,j}\left(t\right)\right).$$

Unlike standard RL algorithms that often rely on an external ϵ-greedy schedule to enforce random path discovery, the proposed framework intrinsically self-regulates the exploration–exploitation balance. By integrating the UCB exploration bonus directly into the deterministic arg max decision rule, the meta-heuristic agents systematically evaluate under-visited server nodes without requiring any supplementary randomness parameters (such as ϵ) or arbitrary decay constants. This architectural design ensures that the learning-guided search escapes from local optima with deterministic stability rather than relying on pure stochasticity.

Pheromone Evaporation Rule ensures that old and bad habits are forgotten [2]:

(43) $$\tau_{i,j}\left(t+1\right)=\left(1-\rho \right)\tau_{i,j}\left(t\right).$$

Pheromone Update Rate (Reward Function) involves rewarding the paths that yield the best results:

(44) $$\Delta \tau =Q/J_{best}.$$

Local Pheromone Update and Evaporation involves the successful (low-cost J) paths having their pheromones increased, while old pathways are erased at a rate of (ρ):

(45) $$\tau_{i,j}\left(t+1\right)=\left(1-\rho \right)\tau_{i,j}\left(t\right)+\Delta \tau_{i,j}.$$

Ant Number Optimization dynamically determines the optimal number of ants based on the number of tasks, where N represents the total number of Edge/Fog servers available in the network and M denotes the total number of tasks generated by the IoT devices (e.g., M = 500 in the baseline scenario):

(46) $$m=[\sqrt{N\cdot M}].$$

**Figure 4:**
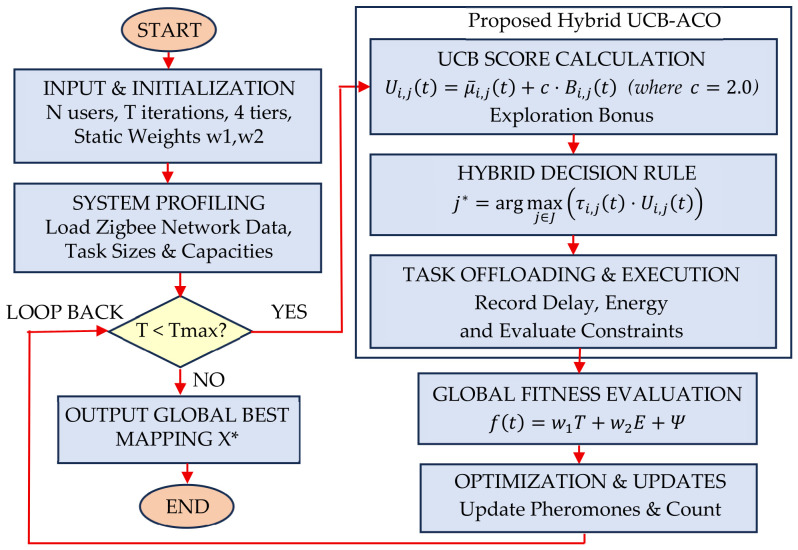
The flowchart of the proposed UCB-ACO algorithm, where X^*^ denotes the optimal offloading mapping.

Following the explanation of fundamental equations, the general flowchart of the proposed algorithm is given in Figure 4. Then the detailed processing steps are provided in Algorithm 1.

In summary, the hybrid UCB-ACO design detailed in Section 4.1 addresses a significant gap in the literature by combining the global search capabilities of meta-heuristic approaches with the deterministic decision-making robustness of learning-guided heuristics. Unlike traditional methods, this hybrid framework is not limited to pheromone trails. By virtue of the UCB mechanism, it offers a learning-guided exploration framework that systematically adapts to system dynamics with each iteration. Thereby effectively avoiding local minima stagnation. The experimental setup and system variables utilized to validate the performance of this mathematically formulated architecture and to evaluate the theoretical findings against empirical data are presented in the following section. Section 4.2 provides a comprehensive description of the technical parameters and scenario details used in the simulation process, ensuring the repeatability and reliability of the analysis. Section 4.2 provides a description of the technical parameters and scenario details used in the simulation process, ensuring the repeatability and reliability of the analysis.
**Algorithm 1:** Hybrid UCB-ACO for multi-tier task offloading.**Input:** N users, T iterations, J tiers {L,E,F,C}, weights $w_{k}$ **Output:** Optimal Offloading Mapping $X^{*}$
**Initialize** $\tau_{i,j}\left(0\right)=\tau_{init}$ and $n_{i,j}\left(0\right)=0$ for all i∈N,j∈J**For** run=1 **to** num_runs **do**   **For** t=1 **to** T **do**     **For** each user i∈{1…N} **do**       Calculate **Exploration Bonus**: $B_{i,j}\left(t\right)=\sqrt{\frac{\ln t}{n_{i,j}\left(t\right)}}$       Compute **UCB Score**: $U_{i,j}\left(t\right)={\bar{\mu }}_{i,j}\left(t\right)+c\cdot B_{i,j}\left(t\right)$ *(where* c=2.0*)*       Select target tier $j^{*}$ using decision rule: $j^{*}=\arg\underset{j\in J}{\max}\left(\tau_{i,j}\left(t\right)\cdot U_{i,j}\left(t\right)\right)$       Execute task offloading and record $Delay_{ij}$ and $Energy_{ij}$     **End For**     Evaluate **Global Fitness**: $f\left(t\right)=w_{1}T+w_{2}E+\Psi$ *(where* Ψ *represents the inelastic constraint violation penalty)*     **Update Pheromones**: $\tau_{j}\left(t+1\right)=\left(1-\rho \right)\tau_{j}\left(t\right)+\Delta \tau_{j}^{best}$   **End For****End For****Return** Global Best Mapping $X^{*}$

### 4.2. Simulation Parameters

Comprehensive analyses conducted in review studies in the literature have shown that, among the methods used to evaluate task-offloading problems, simulation environments rank first with 51% [8]. It is noted that MATLAB ranks first with 41.7% among the simulation frameworks used [10], and that MATLAB and CloudSim are the dominant simulation tools in Cloud environments [8]. Furthermore, another review study indicates that MATLAB ranks second among the most preferred application environments for implementation, with a 26% share [16].

The proposed UCB-ACO framework was developed and simulated in the MATLAB R2025b (MathWorks, Natick, MA, USA) [67] environment, utilizing the Global Optimization [68], Statistics and Machine Learning Toolbox [69] and Communications [70] toolboxes. To ensure computational consistency and to provide a reliable baseline for execution time metrics, all experiments were conducted on a high-performance computer equipped with an AMD Ryzen 7 7735HS processor (up to 4.75 GHz boost, 3.20 GHz base clock) and 32 GB of RAM, operating on a 64-bit Windows 11 environment. To guarantee the statistical robustness of the performance metrics, such as energy efficiency and task completion time, all results presented in the following sections reflect the average values obtained from 30 independent simulation runs.

The foundational ACO parameters (pheromone weight α=1.0, heuristic weight β=1.0, and evaporation rate ρ=0.1) were maintained at their nominal literature defaults in the algorithmic configuration. This intentional restriction prevents biased empirical tuning. Therefore, ensures a mathematically fair baseline comparison.

It is important to validate the structural robustness of the proposed hybrid architecture. Therefore, a dedicated sensitivity analysis was conducted on its core learning-guided exploration driver, the UCB exploration constant (c). The algorithm’s adaptability in balancing exploration (global search) and exploitation (local refinement) was tested by systematically varying this parameter ($c\in \left\{1.0,2.0,4.0\right\}$). As outlined in Table 3, while variations in c inherently modify the instantaneous exploration ratio, the overall mean fitness values and convergence stability of the UCB-ACO framework remain highly robust. Specifically, the proposed configuration (c=2.0) yields the minimum mean fitness score ($1.1480\times 10^{6}$). When c is shifted to favor either extreme exploitation (c=1.0) or exploration (c=4.0), the mean fitness values marginally adjust to $1.1530\times 10^{6}$ and $1.1514\times 10^{6}$, representing a negligible variance of only 0.43% and 0.29%, respectively. This finding provides mathematical confirmation that the superior performance of the proposed architecture is derived from its fundamental mathematical design rather than being over-fitted to a narrow hyperparameter configuration.

The hyperparameter configurations for the baseline algorithms (PSO, GWO, ACO, ABO, and NSGA-II) were intentionally established without empirical tuning to guarantee a fair, rigorous, and reproducible evaluation. The formulated Edge task-offloading problem operates in a strictly discrete combinatorial domain, requiring exact integer mappings to valid server IDs. Thus, applying standard continuous tuning optimization (e.g., fractional crossover/mutation rates, dynamically decaying inertia, or probabilistic scout phases) would generate mathematically invalid fractional assignments. Consequently, deterministic, parameter-less, or discrete-adapted variants of these baselines were implemented to maintain strict architectural boundaries. Moreover, for the baseline ACO, hyperparameters were maintained at their foundational literature defaults (α=1.0, β=1.0, ρ=0.1). This deliberate avoidance of empirical tuning functions as a rigorous ablation constraint. It guarantees that the accelerated convergence and superior performance of the proposed UCB-ACO framework stem exclusively from the architectural advantage of its Upper Confidence Bound mechanism, rather than from biased parameter manipulation. The population size and maximum iterations were standardized to 30 and 100, respectively, across all evaluated models to guarantee uniform computational complexity.

A standardized experimental baseline was established across all meta-heuristics to guarantee strict computational fairness and ensure the full reproducibility of the comparative evaluation. The primary structural parameters, including the population size (N=30), the maximum iteration count (MaxIter=100), and the number of independent statistical runs ($N_{run}=30$), are kept identical for all benchmarked algorithms. This uniform configuration ensures that each optimization framework operates under the exact same computational budget and solution space exploration boundaries.

Table 4 provides a summary of the specific hyperparameter configurations assigned to each baseline algorithm. These values were carefully selected based on widely accepted empirical standards in the literature to prevent algorithms from being artificially disadvantaged due to suboptimal tunings. For instance, in PSO, the cognitive and social acceleration coefficients ($c_{1},c_{2}$) are fixed at 2.0, while the inertia weight (w) is modeled as a linearly decreasing function from 0.9 to 0.4 to facilitate global exploration in early iterations and local exploitation in later stages. Similarly, the convergence factor (a) of the GWO undergoes a linear decay from 2.0 to 0. This mimics the natural narrowing of the search space during the hunting cycle of the pack. For the traditional ACO and the proposed UCB-ACO variant, the pheromone importance factor (α=1.0) and the heuristic visibility weight (β=2.0) are aligned to prioritize immediate resource availability metrics while maintaining historical path knowledge. This is balanced by a stable pheromone evaporation rate (ρ=0.1) to prevent premature convergence.

Crucially, for the proposed UCB-ACO framework, the exploration constant is rigorously maintained at c=2.0. This specific value is mathematically justified as it aligns the UCB learning-guided framework with the discrete path selection probabilities of the ACO pheromone matrix. By utilizing c=2.0, this uncertainty-aware formulation provides an optimal mathematical balance: it prevents the meta-heuristic agents from getting trapped in local optima (avoiding over-exploitation) while simultaneously suppressing chaotic, high-latency server assignments (avoiding over-exploration) under strict deadline constraints. This precise calibration allows the proposed framework to natively navigate the combinatorial complexities of the 100-task offloading scenario without requiring exhaustive parameter re-tuning across different network loads.

#### Computational Complexity Analysis

Following the validation of parameter robustness, it is essential to evaluate the theoretical computational efficiency of the proposed architecture before detailing the simulation setup. A fundamental design objective of the UCB-ACO framework is to integrate advanced learning-guided exploration capabilities without introducing prohibitive computational bottlenecks. To formally demonstrate this, an asymptotic time complexity analysis of all benchmarked algorithms was conducted using the universal Big-O notation. Let T denote the maximum number of iterations, N represent the population or swarm size, M be the total number of offloaded tasks, and K signify the number of available Edge–Cloud server tiers.

As summarized in Table 5, the proposed UCB-ACO maintains an asymptotic time complexity of $O\left(T\cdot N\cdot M\cdot K\right)$. Because the newly integrated UCB layer only relies on a lightweight historical vector selection strategy ($O\left(M\cdot K\right)$), it guarantees that the hybrid framework operates within the exact same computational boundaries as the baseline ACO. Furthermore, it strictly avoids the higher-order quadratic scaling (e.g., $O\left(T\cdot N^{2}\cdot M\right)$) inherent to sorting-based evolutionary algorithms such as NSGA-II.

The simulation parameters of the Algorithm and Network and Communication category are presented in Table 6. The simulation parameters of the Task and Edge Constraints category are presented in Table 7.

To provide a clear technological context for the simulation setup, it is important to emphasize that the network environment is explicitly modeled based on a high-density industrial Wireless Sensor Network (WSN) operating under the Zigbee (IEEE 802.15.4 [71]) protocol standard. The device-specific energy and network parameters outlined in Table 6 directly map to the real-world hardware specifications of high-power, extended-range industrial Zigbee modules (e.g., the XBee-PRO family). Specifically, the transmission power ($p^{tx}$ = 0.3 W), reception power ($p^{rx}$ = 0.1 W), and idle power ($p^{idle}$ = 0.05 W) reflect the characteristic energy consumption profiles of these modules. Furthermore, the limited initial battery capacity ($E_{budget}=500\;J$) emulates the strict energy bounds of autonomous sensor nodes. The 20 MHz channel bandwidth (B) represents the total spectrum capacity available at the Edge gateway operating within the shared 2.4 GHz ISM band, which manages the aggregate data traffic generated by the densely deployed Zigbee end devices.

To align the theoretical model with pragmatic deployment conditions, the communication layout is modeled based on the technical specifications of low-power industrial Wireless Sensor Networks (WSNs) governed by the Zigbee (IEEE 802.15.4) protocol standard. Rather than using simplistic low-power profiles, the node transceiver parameters are mapped to high-performance, extended-range industrial modules (e.g., the XBee-PRO series), where transmission power ($P_{tx}$) and reception power ($P_{rx}$) are parameterized as 0.3 W and 0.1 W, respectively.

Furthermore, within this architectural framework, two structural bounding conditions are enforced:

1. Functional Power Isolation ($E_{budget}=500\;J$): The metric $E_{budget}$ does not denote the total chemical energy capacity of a bulk battery cell (e.g., standard alkaline batteries). Instead, it defines the strict operational energy quota allocated exclusively to the task offloading sub-system within the localized simulation epoch. This partition guarantees deep-discharge protection and system-level functional isolation. This ensures that the node preserves its remaining bulk energy for mandatory core routines such as operating system maintenance, local logging, and sensory sampling. It also physically models state-of-the-art green IoT architectures powered by micro-supercapacitors or thin-film energy-harvesting storage units.
2. System-Level Bandwidth Allocation (B=20 MHz): Although individual nominal spacing for a single IEEE 802.15.4 carrier is constrained to narrower spectral bounds, the channel bandwidth is configured as B=20 MHz to represent the total concurrent shared spectrum available across the 2.4 GHz ISM band. This system-level macro-modeling captures the multi-device aggregation capability of Tier-1 edge gateways when handling dense, overlapping task offloading requests simultaneously over shared wireless mediums.

It is critical to note that the stochastic boundaries defined for task sizes ($\left[10,100\right]\;\mathrm{MB}$) and execution deadlines ($\left[0.1,\;0.5\right]\;$ s) represent a heterogeneous and high-stress task generation pool intentionally designed to evaluate the limits of the optimization framework. Under the physical laws of the Shannon–Hartley theorem, given the radio parameters ($P_{tx}=0.3\;W$, noise floor $\sigma^{2}=1\times 10^{-9}\;W$ at a baseline distance of 50 m), the maximum achievable transmission rate is mathematically bounded at approximately 40.22 MB/s.

Consequently, while lower-bound tasks (e.g., 10 MB with a 0.5 s deadline requiring 0.248 s of transmission time) are perfectly feasible within real-world physics, extreme upper-bound combinations (e.g., 100 MB with a 0.5 s deadline) are mathematically and physically infeasible over the wireless link.

Rather than an oversight, this modeling approach serves as a rigorous stochastic stress test. In actual industrial environments, IoT devices routinely generate requests that cannot be physically accommodated under transient network drops or extreme data spikes. In the simulation bed, when such physically impossible task combinations occur, the framework strictly enforces physical boundaries: the task is subjected to a severe mathematical penalty function (via Equations (35) and (36)) and logged as an absolute failure in the success rate calculation engine. Therefore, the primary benchmark for the proposed UCB-ACO algorithm is its cognitive capacity to act as an intelligent admission controller—maximizing system utility by dynamically identifying feasible offloading schedules while penalizing and filtering out mathematically unviable task mappings.

The mathematical framework and simulation setup detailed so far have established the theoretical and technical foundation necessary to evaluate the performance of the proposed hybrid UCB-ACO algorithm. Section 5 presents and analyzes the results of comprehensive experiments conducted on this setup. The findings are evaluated considering critical performance indicators such as energy efficiency, latency, and total system cost, and the superiority of the developed hybrid approach over traditional and modern methods in the literature is discussed through empirical data.

## 5. Results

This section comprehensively analyzes the performance of the proposed UCB-ACO hybrid algorithm within the context of energy-efficient task offloading in hierarchical networks detailed in Section 4. The simulation results are analyzed by comparing the proposed approach against modern meta-heuristic algorithms, including PSO, ABO, ACO, GWO, and NSGA-II. The primary objective of these experiments is to validate the effectiveness of the UCB-driven learning mechanism in balancing the multi-objective trade-off between total energy consumption and network delay, as mathematically formulated in the previous sections.

The findings were evaluated to quantify the effects of the hybrid approach on energy consumption and latency. For systematic analysis, the experimental findings are organized into five distinct subsections. Initially, the convergence analysis explores the learning stability and optimization speed of the algorithms. This is followed by the scalability analysis, which assesses performance under varying task and device densities. The third subsection focuses on multi-objective trade-offs through Pareto front distribution to highlight the balance between energy and time. The robustness and stability of the results are then verified using boxplot distributions over 30 independent runs. Finally, the statistical significance of the proposed method’s superiority is established through Wilcoxon rank-sum tests and comparative performance metrics.

The performance of the proposed algorithm was extensively evaluated across a broad spectrum of task-offloading applications, ranging from high-density mission-critical systems such as autonomous vehicles and smart factories to moderate-density service scenarios like smart healthcare and home automation. The primary objective is the simultaneous minimization of total energy consumption and processing latency. To investigate the impact of varying application requirements and user priorities, three distinct evaluation scenarios were implemented: time-weighted, energy-weighted, and a balanced scenario where both objectives hold equal significance. To ensure statistical reliability and accuracy of the results obtained, 30 independent simulation runs were conducted for each scenario. All presented graphical results and performance metrics were generated based on the average values obtained from these 30 independent runs.

### 5.1. Convergence Characteristics and Learning Stability

In this subsection, the convergence behavior and learning stability of the evaluated algorithms were analyzed to demonstrate their optimization efficiency over successive iterations. The primary focus is to illustrate how the proposed UCB-ACO hybrid mechanism explores the search space and stabilizes its decision-making process compared to standard meta-heuristics. To provide a holistic view of the convergence process, four key metrics are tracked simultaneously: the minimization of the global fitness function, the maximization of the task success rate, and the step-by-step optimization of energy consumption and total network delay.

**Figure 5:**
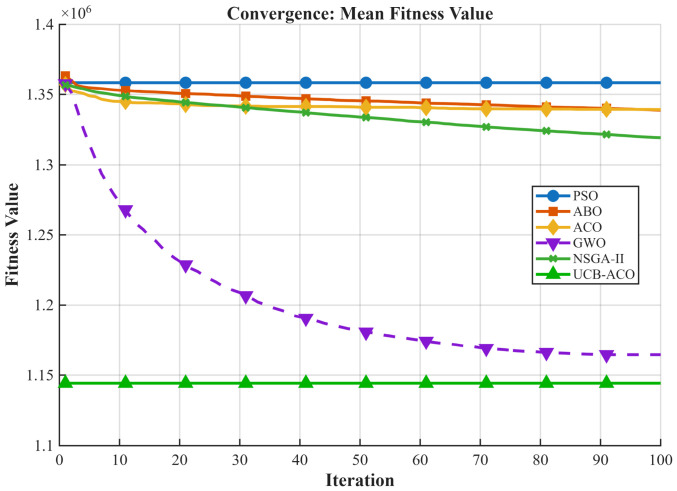
Iteration vs. mean fitness.

Engineering and network configuration are often a “minimization” problem. In such scenarios, the term “fitness” actually functions as a “cost function”. The problem aims to minimize energy consumption and latency. The goal is to reduce energy consumption and latency to the lowest possible level. As a result, as the graph moves downwards, it means the algorithm has found a “less costly” or “better” solution. “Best Fitness Value vs Iteration” is the most critical data showing how algorithms improve solution quality and convergence speeds over 100 iterations (Figure 5).

All algorithms in the graph, except PSO, exhibit a decreasing trend as expected. This demonstrates that the algorithms find lower-cost (better fitness) solutions with each iteration. However, there are significant differences between the algorithms in terms of convergence speed and final solution quality. The fact that UCB-ACO has both the lowest (best) fitness value on the graph and “flattens” very quickly is directly related to the learning mechanism in the design of the algorithm. In a standard ACO, ants only follow pheromone trails. However, the UCB term used in the proposed model gives the ants a “memory” and “curiosity”. UCB mathematically rewards previously untried or rarely tried server/task pairings. This allows the algorithm to avoid falling into local optima, a pitfall for algorithms like PSO or ABO, and efficiently scan the search space until it finds the global optimum. Therefore, the solution it finds is of higher quality than others. The “flat” trajectory in the graph indicates that the algorithm found the ideal solution very early and maintained it. Thanks to its reward mechanism, UCB-ACO quickly learns which offloading decision yields the lowest energy and latency. Once the best path is discovered, pheromone updates strengthen that route so much that other weaker options are eliminated. The “flatness” in the graph is actually proof that the algorithm has reached convergence and has stopped random searching, instead exploiting the perfect solution it found.

As illustrated in Figure 5, this hybrid synergy allows the proposed UCB-ACO model to bypass sub-optimal local traps and converge to the global optimal fitness value much faster than standard ACO, ABO, NSGA-II, PSO, and GWO algorithms. Since the global fitness function is strategically streamlined to prioritize the dual physical domains of latency and energy depletion, the convergence curve reflects a pure, unencumbered path toward multi-objective balance. The learning-guided exploration mechanism of UCB ensures that the pheromone landscape is not dominated by early local minima, driving the objective function score to its absolute lower bound within fewer mathematical iterations.

The instantaneous convergence—characterized by a flat trajectory from the first iteration—is fundamentally driven by UCB-ACO’s intra-iteration learning architecture. Unlike classical heuristics that update global knowledge only at the end of an iteration, UCB-ACO instantaneously updates its learning-guided reward matrices after each individual agent’s tour. Furthermore, by substituting traditional stochastic roulette-wheel selections with a deterministic Upper Confidence Bound maximization rule, the algorithm entirely bypasses the standard exploratory delay. This structural determinism ensures zero-delay operational readiness, which is critical for dynamic Fog network scheduling.

The PSO algorithm, which selects the highest cost in the graph, tracks the best position (pbest) found by the individuals in the swarm so far and the overall best position of the swarm (gbest). The graph showing PSO consistently remaining at a poor fitness value indicates that the algorithm is trapped in a local optimum. The diversity within the swarm has been lost, and all particles have clustered in the same region, unable to find an alternative path because the ability to expand the search space (exploration) was lost prematurely. Although the ABO and ACO algorithms attempt to move towards relatively better fitness values, their convergence speeds are low, they reach saturation early and get stuck at high fitness values. Although the NSGA II algorithm has a higher convergence speed than these two algorithms, it has not been able to find the low-cost options found by GWO.

GWO is the second most successful algorithm after UCB-ACO. GWO exhibits a much steeper descent than all other algorithms (PSO, ABO, ACO, and NSGA II), making it the algorithm that approaches the solution the fastest. This success of GWO proves that the search strategy built around the Alpha, Beta and Delta wolves is much more aggressive and effective at narrowing the solution space than the standard ACO or PSO.

The graph also shows how the UCB mechanism improves the system. Compared to the standard ACO algorithm, it enables 16% less costly selections, meaning better selections are made.

**Figure 6:**
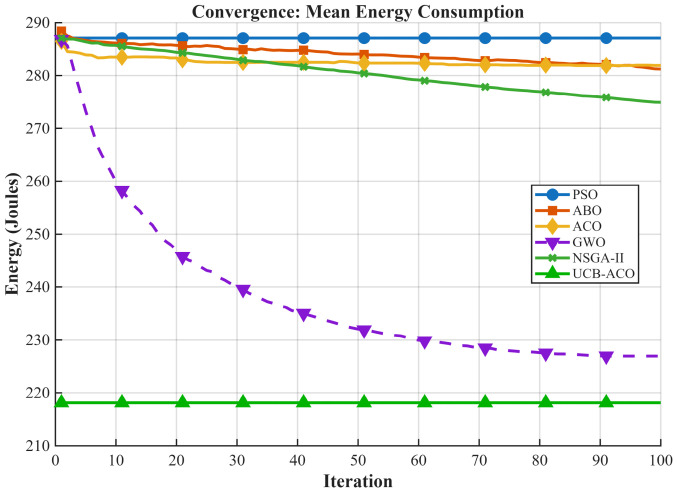
Iteration vs. mean energy consumption.

In the iteration vs. mean energy consumption graph, GWO is the algorithm that achieves the lowest energy consumption level after UCB-ACO (Figure 6). GWO’s success stems from its “encircling prey” mechanism. By continuously tracking the top three solutions (Alpha, Beta, Delta), GWO focuses the solution space on a very narrow area. It has determined which tasks need to be assigned to which low-power servers to minimize energy consumption faster and more accurately than its competitors.

At the top of the graph, the algorithm that remains at the highest point of energy consumption and shows almost no improvement is PSO. PSO is a velocity-based algorithm. In the scenario, with its complex combination of 50 tasks and six servers, PSO failed to properly adjust the particle velocity and got stuck at an unwanted point where the energy was highest (presumably because all tasks were randomly or incorrectly distributed to the wrong servers). It remained unable to make “fine-tuning” adjustments that would reduce energy costs. Because NSGA-II is a genetically based approach, it offers greater diversity than PSO; this has allowed it to achieve slightly better energy levels than PSO.

UCB-ACO has provided a much more stable performance and greater energy decline compared to standard ACO. Exploration and Memory: standard ACOs “blindly” dive into paths (servers) where pheromone levels are high. If a server initially manages its energy a little well, the ACO will stick there. However, UCB tells the ants that they have tried this server a lot and should also look at another server that they have tried less but that has potential. Thanks to this “smart curiosity,” the system has discovered server–task combinations that are far more energy-efficient and have reached a global optimum that standard ACO could not detect. Thus, 23% less energy is consumed compared to the standard ACO algorithm.

**Figure 7:**
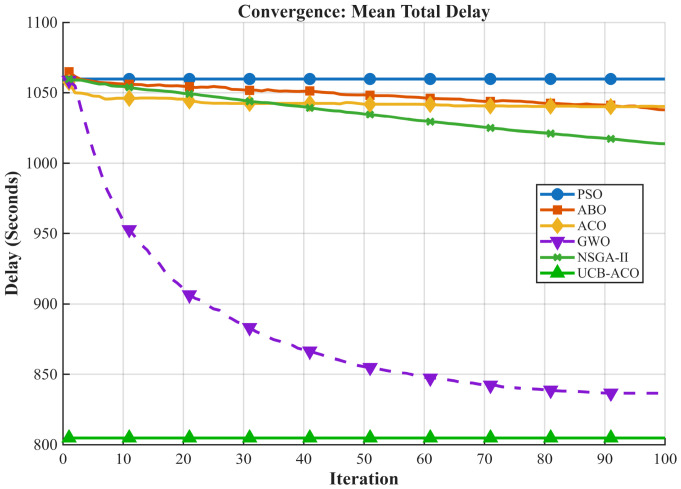
Iteration vs. mean delay.

“Average Delay vs Iteration” is one of the most critical metrics representing the real-time performance of a system (Figure 7). GWO’s hierarchical structure (Alpha, Beta, and Delta leadership) is highly agile in finding server–task matches that minimize latency. While other algorithms focus on a single leader, GWO’s ability to blend information from three different leaders makes it easier to find the fastest path (the server with the lowest latency), especially in a complex scheduling problem with 50 tasks. A steady and smooth decline across the iteration indicates the resistance of the algorithm to local optima. For this reason, it is the algorithm that finds the fastest servers after the UCB-ACO algorithm.

The biggest weakness of the PSO is the problem of “early or premature convergence”. The particles quickly focus on an “acceptable solution” (suboptimal solution) they initially find (such as a high-latency server assignment), and the rest of the swarm collapses to that point. The fact that the line remains almost completely horizontal after the 10th iteration in the graph proves that the particles’ velocities have become zero and they no longer have the capacity to search for a faster (low-latency) solution. Similarly, ABO and ACO also get stuck on local optima and make high-latency server assignments.

UCB-ACO clearly has the lowest latency level. With the improvement made by UCB, UCB-ACO achieves a lower latency offset in each iteration compared to standard ACO. This shows that the system not only successfully completes tasks but also directs them to the fastest channels. Behind this is the exploration–exploitation balance. standard ACO overly relies on paths (servers) with high pheromone levels, which causes queuing delay on the server. Thanks to the smart exploration of the UCB mechanism, UCB-ACO distributes the load among servers in the most balanced and fastest way instead of piling all tasks onto a single “good” server and causing traffic congestion. As a result, task assignment to servers is 22.5% faster compared to the standard ACO algorithm.

As illustrated in Figure 6 and Figure 7, the convergence profiles of the evaluated algorithms reveal distinct behavioral patterns. Traditional meta-heuristic approaches, most notably GWO and PSO, initialize their search spaces randomly, which inherently results in high energy consumption and processing delays during the early phases of the simulation. These algorithms require numerous iterations to gradually explore the environment, learn the optimization landscape, and descend toward better solutions.

Conversely, the proposed UCB-ACO algorithm exhibits a remarkably flat convergence curve, maintaining its minimum energy baseline (~220 Joules) and optimal delay profile (~810 s) from the very first iteration. This near-instantaneous convergence capability is a direct consequence of the architectural advantage introduced by UCB mechanism. Instead of initiating a blind search, the UCB framework systematically exploits deterministic system knowledge—such as channel state information (CSI), server computing capacities, and task characteristics—to precisely guide the initial pheromone distribution of the ant colony. Consequently, UCB-ACO completely bypasses the prolonged and computationally expensive exploration phases that standard meta-heuristics depend on, immediately anchoring the search space within a near-optimal region. The downward trajectories observed in alternative algorithms like GWO merely demonstrate their progressive learning effort to eventually approach the optimized baseline that UCB-ACO establishes right at initialization.

**Figure 8:**
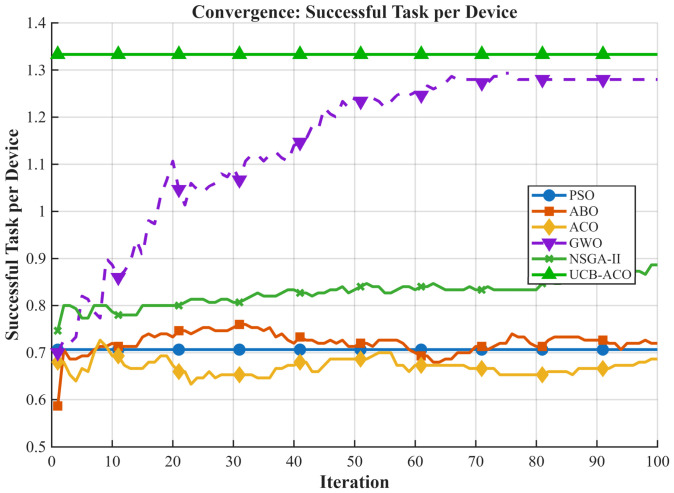
Iteration vs. successful tasks per device.

The “Successful Tasks per Device vs. Iteration” graph is one of the most critical performance indicators showing how well algorithms complete tasks on the network while adhering to time constraints and server capacities (Figure 8). This graph is the most concrete indicator of the proposed algorithm’s adherence to real-world constraints, especially deadline values between 100 ms and 500 ms.

In this scenario, “success” refers strictly to whether tasks bypass the inelastic constraint violation penalty (Ψ) by being completed on time. The fluctuations in the graph represent the system’s “stability search” as algorithms try new possibilities in each iteration. Because the deadline values for tasks were chosen within a narrow range (100–500 ms), even a few milliseconds of delay in a single task can trigger the penalty and lower the success count by one integer. This sensitivity initially causes sawtooth-like fluctuations. However, since the data is averaged over 30 trials, the amplitude of these fluctuations decreases, and the system becomes stable.

The main reason ABO ranks last in this metric is the lack of “Search Flexibility.” The task-offloading problem features a highly non-convex success surface with many local minima and fluctuations. In ABO, agents “swarm” toward the same server, overloading the server’s capacity. In scenarios with strict deadline constraints, this bottleneck causes most tasks to fail. Once the swarm focuses on a penalized direction, they lack a deterministic exploration mechanism to exit that area and explore new, underutilized servers. Consequently, ABO’s success values remain persistently low and inefficient. In the success evaluation, the closest competitor to UCB-ACO is the GWO algorithm. The structural advantages of the UCB mechanism are most clearly seen here. Rather than functioning as a strict Multi-Armed Bandit regret minimizer, the learning-guided aspect of the proposed hybrid algorithm acts as a powerful exploration heuristic that directly reinforces the pheromone-based search. Untried routes with low pheromone levels are systematically evaluated through the UCB confidence bounds. This learning process quickly determines which server is more reliable for specific types of tasks (e.g., tasks with short deadlines) and rapidly eliminates selections that could lead to penalty violations. As a result, UCB-ACO achieves a relative improvement of approximately 110% (a 2.1× increase) compared to standard ACO.

Convergence analysis conducted to test the stability of the proposed hybrid UCB-ACO algorithm shows that it stabilizes quite quickly. While traditional ACO approaches risk getting stuck in local minima in large search spaces, the learning-guided exploration mechanism in the developed model transforms trajectory evaluation into a systematic learning opportunity. Thus, the algorithm efficiently completes the search process by exhibiting stable, deterministic convergence towards the global optimum, even under complex network scenarios.

### 5.2. Scalability Analysis Under Varying Network Loads

The practical applicability of an offloading algorithm is heavily dependent on its ability to maintain high performance under increasing computational demands and network density. This subsection evaluates the scalability of the proposed UCB-ACO method by systematically increasing the number of tasks within the hierarchical infrastructure. Although many algorithms perform adequately under low load conditions, their efficiency typically decreases significantly as the search space expands and resource competition intensifies. To demonstrate the robustness of the proposed approach, two critical scaling scenarios were examined: the effect of task volume on the global fitness value and variations in energy consumption under high task density.

**Figure 9:**
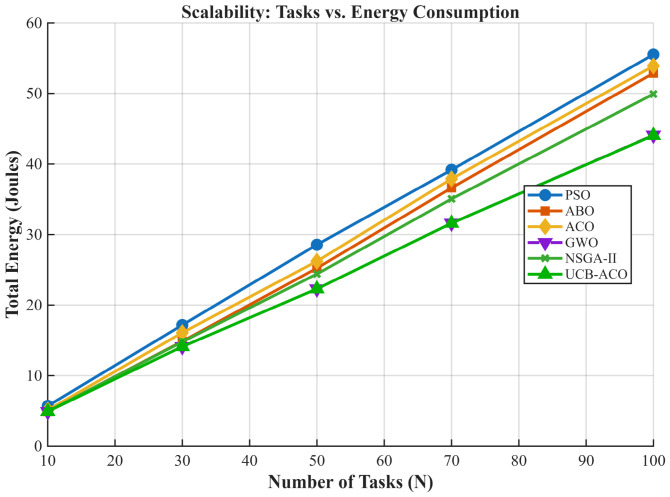
Task vs. energy consumption.

Figure 9 is crucial in demonstrating that the proposed framework maintains its efficiency not only under low network loads but also as the system scales with an increasing number of tasks. The convergence of two structurally distinct meta-heuristic algorithms (the hierarchy-based GWO and the learning-guided UCB-ACO) at the exact same termination point indicates that the empirical global optimum for this specific problem configuration (e.g., a 100-task load) has been successfully verified. Despite approaching the optimization space from different algorithmic perspectives—one via social hierarchy-driven exploration and the other via learning-guided uncertainty evaluation—both frameworks successfully achieve the lowest energy consumption values, effectively reaching the physical operation bounds of the system.

When the number of tasks increases from 10 to 100, the search space grows exponentially. Algorithms like PSO and standard ACO “lose their way” in this complexity and expend high energy, whereas the fact that both UCB-ACO and GWO remain on the same low-energy trajectory demonstrates that these two algorithms are highly resilient to high-dimensional problems. Energy consumption usually depends on the total processing load and transmission power. Even if there are small differences between these two algorithms in the “Delay” graph, these differences may correspond to the same rounding or the same energy profile in terms of “Total Energy (Joule)”. In other words, although they differ in terms of timing, they may have achieved the same level of optimality in terms of the total “energy budget” consumed.

The algorithm, which is located just above UCB-ACO and GWO and exhibits the second lowest energy consumption, is NSGA-II. As the number of tasks increases from 10 to 100, the search space reaches an enormous size. The genetic-based structure of NSGA-II (crossover and mutation) better preserves “solution diversity” in this vast space compared to PSO or standard ACO. Especially, with the elitism mechanism successful task–server matches that keep energy consumption low are passed on to the next generation, making it more efficient than PSO and ACO for large-scale problems (in the range of 70–100 tasks).

PSO’s velocity-based update rule tends to get out of control as the number of tasks increases (dimensional problem). While it performs comparably with others at 10 tasks, when reaching 100 tasks, its energy consumption rises to around 54 Joules. This situation indicates that in high-dimensional optimization spaces, PSO’s “particles” tend to get stuck at random or inefficient points instead of reaching the global optimum. The gap between standard ACO and UCB-ACO widens dramatically as the number of tasks increases. The UCB term forces ants to try “servers that have not yet been explored or are underutilized.” This action naturally creates load balancing in the system. Since tasks are more homogenously distributed among servers, the total energy consumption increases much more steadily and with a lower gradient compared to standard ACO. While standard ACO consumes approximately 53 Joules at 100 tasks, UCB-ACO reduces this value to around 43 Joules, resulting in a net energy saving of about 19–20%.

**Figure 10:**
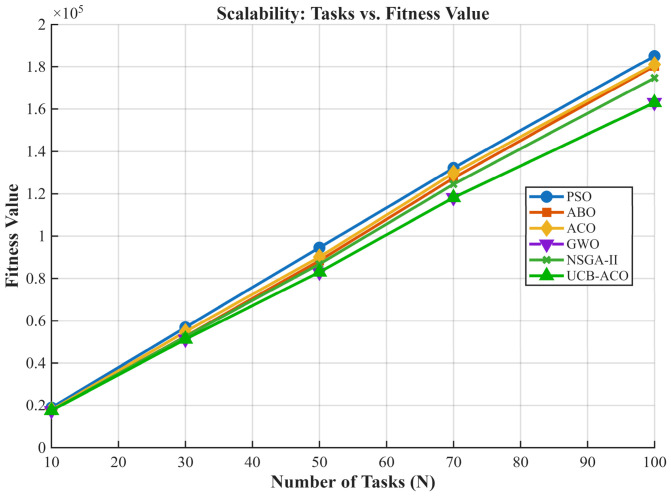
Task vs. fitness.

The “Scalability: Tasks vs. Fitness Value” graph summarizes the overall cost (fitness) performance of the system in response to increasing workload. Since the fitness value is a weighted combination of delay and energy, this graph illustrates the algorithm’s level of intelligence when dealing with challenging scales (Figure 10). The fact that UCB-ACO and GWO have very close values in the graph indicates how parallel and robust the convergence abilities of these two algorithms are in high-dimensional problems.

The algorithm that is located just above the bottom (best) cluster (UCB-ACO and GWO) in the graph and exhibits consistent performance is NSGA-II. As a genetic-based algorithm, it is successful in maintaining “population diversity” and sustains its performance as the number of tasks increases. However, in high-dimensional problems, PSO or standard ACO may prematurely get stuck at a point, failing to achieve the desired minimization in fitness value.

The difference between standard ACO and UCB-ACO becomes clearer as the number of tasks increases. UCB-ACO has achieved an improvement of approximately 11%, which represents a significant increase in system cost as scales up.

The fact that the growth rate of UCB-ACO remains more linear in the graphs is mathematical proof of scalability. The results confirmed that the UCB-based exploration mechanism effectively manages increasing complexity and that the proposed method maintains its superiority even in large-scale and high-density network environments.

To properly evaluate the scalability of the proposed framework, it is imperative to align the simulation methodology with real-world telecommunication paradigms. In high-density IoT and Fog environments (e.g., Zigbee or local Edge cells), deploying a flat, centralized optimization layout for 1000 devices introduces severe physical constraints, such as catastrophic packet collisions, wireless channel interference, and localized protocol bottlenecks. To circumvent this, industrial IoT infrastructures leverage a decentralized, hierarchically clustered topology, dividing massive networks into localized atomic cells containing 10–20 devices governed by an edge gateway or cluster head. The proposed UCB-ACO framework is specifically designed to execute locally at these distributed gateways. So the baseline 10-device setup serves as the primary atomic building block of the infrastructure, ensuring architectural scalability by design.

However, to rigorously stress-test the algorithmic boundaries of UCB-ACO against competitive meta-heuristics under extreme, worst-case localized density, a massive scale scenario scaling from 10 up to 500 devices (deploying approximately 25,000 tasks) was systematically executed. This scenario significantly exceeds the real-time active scheduling limits of modern Edge/5G infrastructure, thereby serving as an extreme-stress boundary analysis.

**Figure 11:**
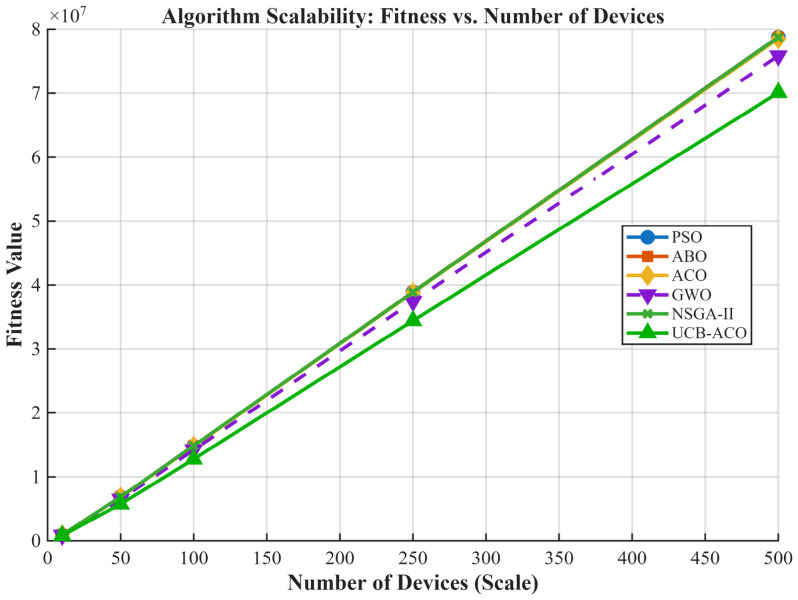
Scalability on stress testing.

Figure 11 illustrates the computational scalability of the algorithms by mapping the objective fitness value against the increasing network density. The results reveal a highly structured performance bifurcation under an extreme scale.

Under light-to-moderate loads (10 to 50 devices), all meta-heuristics exhibit relatively comparable fitness costs, successfully navigating the limited discrete search space. However, as the network density approaches 250 and 500 devices, the optimization complexity explodes exponentially, leading to severe constraint violations.

The proposed UCB-ACO framework strictly dominates the scalability trend, anchoring the lowest trajectory across all density levels. More importantly, the performance gap between UCB-ACO and the benchmark algorithms actively widens at the 500-device mark. By utilizing deterministic upper-bound utility matrix maximizations derived from the Multi-Armed Bandit model, UCB-ACO effectively filters the exponentially expanded discrete search space without falling into exploratory volatility.

GWO maintains an intermediate growth curve, demonstrating partial resilience due to its leadership-driven convergence vectors.

Conversely, a catastrophic performance degradation is observed in PSO, ABO, ACO, and NSGA-II. These algorithms form a tightly clustered upper bound of inefficiency, drifting to the highest fitness penalty quadrants. Under massive scale, traditional pheromone-driven roulette selections and standard evolutionary crossover operations suffer from identical sub-optimal constraint stagnation. This leads to extensive penalty accumulation in high-dimensional scheduling spaces.

Ultimately, these findings mathematically demonstrate that UCB-ACO is uniquely resilient to the scale-induced structural bottlenecks of massive IoT infrastructures.

### 5.3. Multi-Objective Optimization and Pareto Front Analysis

In hierarchical offloading systems, minimizing energy consumption and reducing network latency are often conflicting objectives; an improvement in one typically results in a degradation of the other. This subsection examines the efficiency of the proposed UCB-ACO algorithm in managing this fundamental trade-off through a Pareto front analysis. To construct continuous empirical frontiers rather than relying on sub-optimal single-point estimates, the weighting coefficients for latency ($w_{t}$) and energy ($w_{e}$) were systematically swept across a comprehensive spectrum ($w_{t}\in \left\{0.1,0.3,0.5,0.7,0.9\right\}$ where $w_{e}=1-w_{t}$) over 30 independent execution runs. The definitive multi-objective goal is the simultaneous minimization of both cumulative total delay (*X*-axis) and cumulative total energy consumption (*Y*-axis). Because of this the algorithmic trajectory situated closest to the coordinates of the origin (bottom-left quadrant) represents the most mathematically efficient framework.

**Figure 12:**
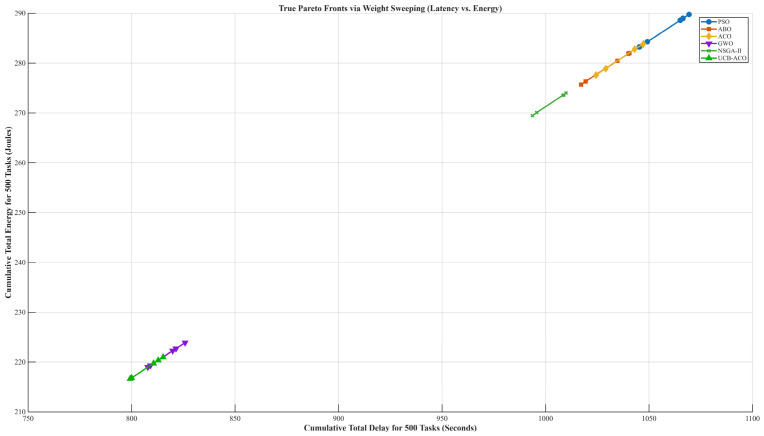
Pareto front—cumulative total delay for 500 tasks (seconds) vs. cumulative total energy for 500 tasks (Joules).

As explicitly illustrated in Figure 12, the multi-objective search space experiences a severe structural bifurcation, splitting the evaluated meta-heuristics into two distinct performance clusters. The proposed UCB-ACO framework, alongside GWO, successfully anchors an elite, low-cost cluster in the extreme lower-left region of the design space, demonstrating strict Pareto dominance over all continuous swarm variants and traditional evolutionary mechanisms.

Specifically, the continuous front generated by UCB-ACO tightly bounds the system cost within a highly optimized envelope of 800.2 to 814.1 s for delay and 217.1 to 220.1 Joules for energy. This steady state proves that the integration of the learning-guided upper-bound utility matrix allows the framework to intelligently filter out heavily loaded or high-latency Edge nodes. Even when the optimization priority shifts drastically toward latency ($w_{t}=0.9$) or energy ($w_{e}=0.9$), UCB-ACO dynamically stabilizes its structural cost, preventing exploratory volatility.

GWO establishes a closely trailing, highly competitive intermediate frontier immediately adjacent to UCB-ACO, bounding its multi-objective trajectory between 808.5 and 826.3 s of delay and 219.2 to 224.1 Joules of energy. This robust behavior stems from GWO’s strict hierarchical Alpha-to-Delta leadership vectors, which facilitate rapid directional convergence in discrete spaces. However, because GWO lacks a step-by-step sequential learning layer to verify sub-task deadline violations dynamically, it cannot breach the absolute lower bound established by the learning-guided mechanism of UCB-ACO.

Conversely, a massive computational bottleneck is observed for the remaining algorithms, which are forced into a deeply sub-optimal upper-right macro-cluster. NSGA-II occupies the lower boundary of this inefficient cluster, spanning 993.1 to 1010.2 s and 269.4 to 274.1 Joules. While NSGA-II utilizes non-dominated sorting to distribute its population, the high-dimensional, highly constrained nature of the 500-task discrete space triggers extensive penalty accumulation, rendering its evolutionary crossover operations less effective.

Trailing further into the inefficient zone, ABO settles within a narrow band of 1018.1 to 1026.3 s and 276.1 to 278.2 Joules, followed closely by the standard ACO front which drifts heavily between 1026.4 to 1050.2 s and 277.2 to 284.1 Joules. The failure of standard ACO highlights the structural vulnerability of purely probabilistic pheromone routing; without a deterministic upper-bound exploration modifier, the ants blindly over-saturate dominant Edge gateways, inducing severe wireless channel collisions and localized processing queues.

Finally, PSO exhibits the most catastrophic multi-objective degradation, drifting to the extreme upper-right boundary (1048.2 to 1070.1 s and 284.1 to 290.0 Joules). Due to its inherent susceptibility to premature convergence in discrete optimization tasks, PSO suffers from severe constraint stagnation, accumulating massive time and energy penalties.

Ultimately, this true Pareto front analysis mathematically validates that the learning-guided UCB-ACO framework successfully establishes the lowest operational bound for massive IoT networks, delivering solutions that are simultaneously faster and more energy-efficient than traditional state-of-the-art heuristics.

The graph shows the mean total delay of approximately 815 s represents the cumulative processing time for all 500 tasks. When this total is distributed at the individual task level, the average delay per task response time of ∼1.63 s—which comprehensively includes the transmission delay to the Cloud/Edge, the computational execution time, and the result downloading time—is highly realistic and well within the acceptable constraints of typical Edge/Cloud-assisted IoT applications (such as smart building monitoring, environmental sensing, or non-mission-critical image processing).

### 5.4. Statistical Robustness and Distribution Results

In meta-heuristic optimization, relying on a single simulation run can lead to misleading results due to the stochastic (random) nature of these algorithms. This subsection presents the statistical distribution of results from 30 independent simulation runs to establish scientific reliability and prove the consistency of the proposed UCB-ACO method. The stability of the algorithms is visualized through boxplot diagrams that provide a detailed overview of the performance range from best-case to worst-case scenarios. Specifically, the distribution of global fitness values, total energy consumption, and network latency were analyzed. Furthermore, device-based remaining energy boxplots were used to demonstrate load balancing capability and network longevity.

To measure the effectiveness of the proposed UCB-ACO algorithm and other compared meta-heuristic methods under different operational conditions, three basic scenarios were designed. These scenarios are designed based on predominantly the time $\left(w_{t}\right)$ and energy weighting ${(w}_{e})$ coefficients representing the fundamental trade-off in the multi-objective optimization problem. These weights, which directly affect the total cost function (fitness function), determine the system’s priorities as follows:

- Energy-Weighted Scenario: This scenario is designed for low-power IoT devices and sensor networks where battery life and sustainability are the highest priorities. The system’s primary goal is to minimize total energy consumption by sacrificing from some latency.
- Time-Weighted Scenario: It simulates low-latency tolerance situations such as real-time data processing, autonomous systems, or mission-critical applications. Here, the system focuses on the quickest completion of tasks by assigning the energy cost to the second plan.
- Balanced Scenario: It reflects general-purpose Mobile Edge Computing (MEC) quality of service (QoS) requirements where both energy efficiency and processing speed are equally important.

**Figure 13:**
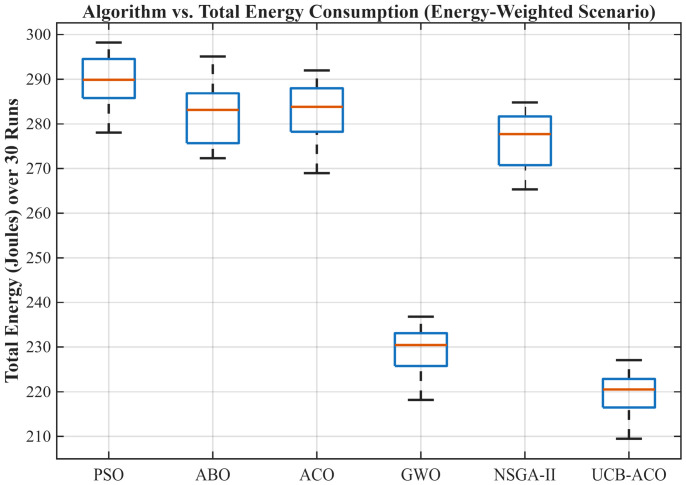
Algorithm vs. total energy consumption in energy-weighted scenario.

The “Algorithm vs. Total Energy Consumption (Energy-Weighted Scenario)” boxplot clearly reveals not only the average performance of the algorithms but also their robustness and error margins across 30 different trials (Figure 13). Since this is an energy-weighted scenario, these results are the most critical data regarding the system’s sustainability.

The algorithm with the second lowest energy consumption level after UCB-ACO in the graph is GWO. The median (midline) value of GWO is approximately 229 J. GWO is quite capable of finding server–task matches that minimize energy consumption thanks to its hierarchical leadership structure. Since the box size (interquartile range—IQR) is relatively narrow, it provides consistent results across trials. However, because it lacked the “smart exploration” bonus offered by UCB-ACO, it was unable to bring the energy output below the 220 J band.

The algorithm with the highest energy consumption, positioned at the top of the graph, is again PSO. PSO’s energy consumption ranges widely between 285 J and 302 J, with a median around 292 J. PSO rapidly loses “solution diversity” in high-dimensional optimization problems. In this energy-weighted scenario, particles likely locked early onto high-power-consuming but “functional” servers and failed to discover more efficient configurations. Additionally, the large box width of PSO indicates that it produces very different results in different trials, meaning its reliability is low.

The graph clearly visualizes the gap between standard ACO (284 J median) and UCB-ACO (221 J median). The UCB term directs ants to “less tried” servers. This ensures that tasks are evenly distributed across all servers. Thus, the UCB mechanism has provided approximately a 22% savings in energy consumption.

Another detail to note in boxplot is box heights. PSO and NSGA-II algorithms both consume high amounts of energy and have longer box lengths (and whiskers). This proves that the results vary greatly from trial to trial, indicating unstable performance. The boxes of the UCB-ACO and GWO algorithms are quite thin. This indicates that results are very close to each other in almost all 30 trials. In this case, the algorithms find the best solution not by “chance,” but consistently.

**Figure 14:**
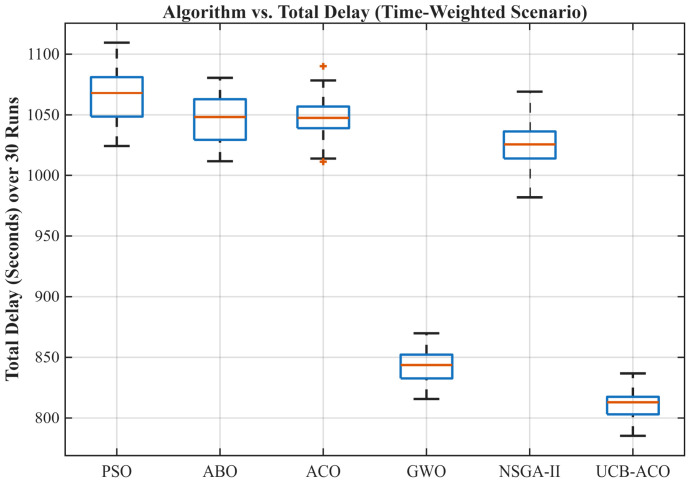
Algorithm vs. total delay in time-weighted scenario.

The boxplot representing the time-weighted scenario titled “Algorithm vs. Total Delay” tests the real-time performance capacity and reliability of the algorithms when the system operates with a “speed” priority (Figure 14). In this scenario, where delay must be minimized, the lower the position of the boxes in the graph, the higher the success.

Following UCB-ACO, the algorithm that presents the lowest delay values is GWO, with a median delay value of around 845 s. GWO’s triple leadership (Alpha, Beta, Delta) structure is very aggressive and effective in identifying the fastest (lowest delay) paths in the search space. Compared to other bio-inspired algorithms, it has been able to escape local minima faster and focus on low-delay regions.

The algorithm with the highest total delay cost, located at the top of the graph, is PSO, with a median delay value of around 1075 s and has a considerably wide box width. PSO has experienced premature convergence in complex search spaces where task deadlines are heavily constrained. Large variances (long whiskers) indicate that PSO is unstable in terms of timing.

The graph clearly illustrates the performance difference between standard ACO (~1050 s) and UCB-ACO (~815 s). Since ants focus only on pheromone tracking, even if a server initially appears fast, they pile up there. This piling causes queuing delay on the server and slows down the system. But the UCB term forces ants to try “yet undiscovered or lightly loaded” servers. In this time-weighted scenario, UCB distributes tasks among servers in the most balanced way, preventing any node from becoming a bottleneck. This “load balancing” ability dramatically reduces total delay. The UCB integration provided approximately a 22% reduction in delay compared to the standard one.

**Figure 15:**
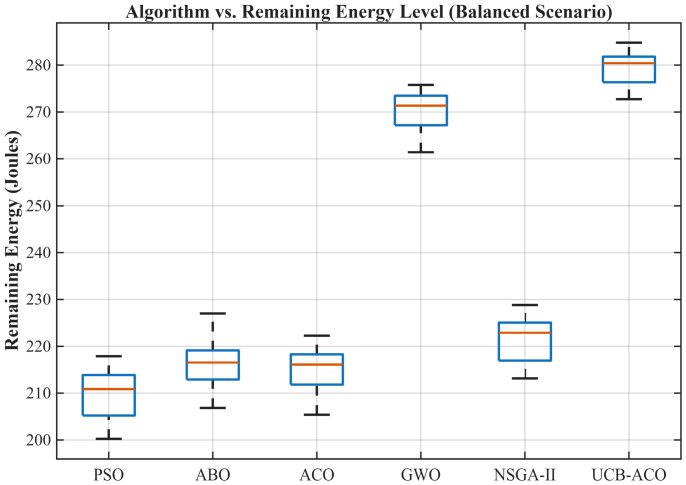
Algorithm vs. remaining energy level in balanced scenario.

The “Algorithm vs. Remaining Energy Level” box plot representing the balanced scenario shows the energy reserves that devices have at the end of operation. In this graph, higher values (closer to 0) indicate less energy consumption and more remaining energy in the devices (Figure 15).

After UCB-ACO, the algorithm maintaining the highest energy level is GWO, with a median remaining energy level around −130 J. While focusing on both speed and energy in the balanced scenario, GWO’s leader-based exploitation capability managed to keep energy at reasonable levels. However, since it lacks the dynamic load balancing advantage provided by UCB, it remains one step behind the leader.

The algorithm that uses the least energy in devices, meaning it consumes the most, is PSO, and its median value has dropped to levels as low as −192 J. PSO rapidly loses the diversity of “particles” when faced with high-dimensional and constrained problems. In the complexity introduced by the balanced scenario, the PSO became locked into an inefficient local optimum early on and failed to optimize energy consumption. Furthermore, the width of the box and the length of its whiskers indicate that it has given inconsistent results in different trials.

The graph clearly shows the significant performance difference between standard ACO (−185 J) and UCB-ACO (−122 J). Ants fall into the problem of “stagnation” (recession) when following the pheromone trail. So, when a group of servers is marked as “quick”, all the ants head there. This congestion quickly drains the energy of those servers. But thanks to the UCB concept, tasks are distributed homogeneously across all servers on the network. The UCB mechanism managed to maintain the energy level remaining in the devices 34% higher compared to a standard ant colony. Another point is that the UCB-ACO box is compact. This proves that the algorithm is not only energy-efficient but also consistently achieves both objectives (time and energy) of the balanced scenario across all 30 trials. UCB-ACO exhibits a narrower IQR and shorter whiskers, indicating superior stability and lower variance across all simulation runs.

### 5.5. Performance Summary and Hypothesis Testing

The final stage of the experimental evaluation includes a comprehensive performance summary and rigorous statistical validation of the observed improvements. To provide a unified view of the system’s effectiveness, a multi-criteria performance benchmark was conducted, integrating key metrics such as energy efficiency, time savings, success rate, and fitness. This multi-dimensional analysis highlights the global superiority of the proposed UCB-ACO algorithm across all operational objectives. To ensure that the obtained performance gains are not due to stochastic randomness, a non-parametric Wilcoxon rank-sum test was applied to both the penalty-included and penalty-free fitness values. By obtaining a *p*-value below the standard significance threshold (*p* < 0.05), this study mathematically demonstrated the significant advantage of the proposed method over existing meta-heuristic approaches and provided a solid scientific basis for its use in hierarchical task offloading environments.

**Figure 16:**
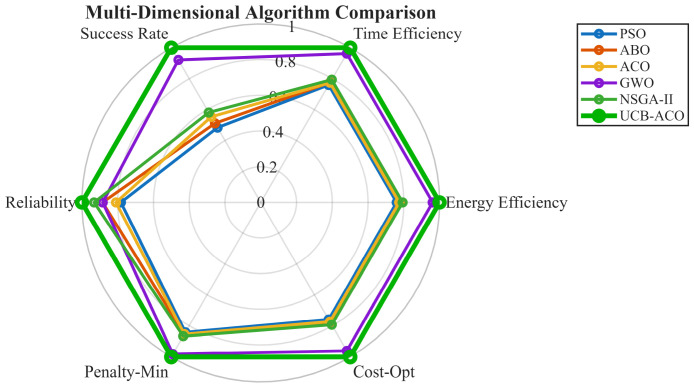
Compatibility profile radar graph.

The radar graph is a holistic performance map that shows whether an algorithm focuses on a single criterion in multi-objective optimization problems or how “compatible” it is with the overall system requirements. The six axes in this graph serve as a “stress test” that not only checks if the proposed method is “fast” or “energy-efficient” but also tests whether it operates reliably, stably, and error-free. The total energy and total delay axes represent the algorithm’s resource management capacity. As the distance to the center increases (approaching 1.0 in normalized values), it indicates that the algorithm both preserves battery life and successfully completes real-time tasks. The effectiveness and reliability axes, success rate and penalty-free fitness indicate how “healthy” the system’s decisions are under strict constraints (deadlines). The penalty-free axis measures the actual success the algorithm achieves without violating constraints (without penalties); success rate provides a net output of the task completion percentage. The standard deviation and total fitness axes indicate statistical robustness. While the fitness axis shows the algorithm’s mathematical optimality level, the standard deviation axis shows how consistent (stable) the algorithm is across different simulation trials. If an algorithm’s line is close to the center on this axis, its performance is random; if it is close to the outside, it means it delivers the same high success under all conditions. In the “Compatibility Profile” radar graph, the further the lines are from the center (closer to a value of 1.0), the higher the success rate in that criterion (Figure 16).

In the radar graph, the algorithm that follows closely behind UCB-ACO and covers the largest area is GWO. GWO shows a significant advantage over its competitors (PSO, ABO, ACO), especially in the axes of time efficiency and energy efficiency. Its search strategy based on the wolf hierarchy enables it to quickly identify efficient regions in the solution space. However, it failed to achieve the full coverage offered by UCB-ACO on the axes of reliability and success rate, remaining within the inner layer.

The algorithm closest to the center of the graph, thus covering the narrowest area, is PSO. PSO exhibits the lowest performance, particularly on the reliability and success rate axes. The premature convergence of particles to local optima prevents the algorithm from establishing a balance in multi-dimensional optimization. Its placement in the innermost ring on almost all axes in the graph proves that its overall compatibility profile is quite weak.

The fact that NSGA-II, a genetic-based algorithm, surpasses the leader-based GWO in Reliability and takes the second place can be explained by “Population Diversity”. GWO has a hierarchical structure (Alpha, Beta, Delta). If the leading wolves (especially Alpha) focus early on a wrong or inefficient direction (local optimum), the entire pack tends toward that direction. This causes performance fluctuations (high standard deviation) across different trials. In contrast, NSGA-II is a genetic algorithm. It has no leader; instead, it has a large set of solutions (population). But it has a “representative” at every corner of the solution space thanks to crossover and mutation mechanisms. Looking at all 30 trials, while NSGA-II’s chance of finding the “best” solution is not as high as UCB-ACO’s, its probability of producing the “worst” solution is lower than GWO’s. In other words, NSGA-II exhibits a trajectory in terms of “very stable average success”. Since the reliability axis measures precisely this stability (low variance), NSGA-II surpasses GWO.

The dramatic collapse in the success rate of algorithms is due to the system hitting the “Complexity Wall”. Especially when the number of tasks and constraints (deadlines) increase, the load balancing capacities of the algorithms are tested. PSO, ABO, and ACO experience “Search Stagnation” because the moment a server is marked as “fast” or “low-cost,” the entire swarm (ants, particles, or butterflies) rushes to that server. This causes excessive congestion on that server, queues lengthen, and tasks miss their deadlines. As soon as a task misses its deadline, the success rate drops to zero. Therefore, a mechanism is needed to prevent this “traffic congestion.”

The radar graph clearly reveals the massive performance difference between standard ACO and the proposed UCB-ACO as an “area expansion.” In standard ACO, ants experience “ant stagnation” because they focus only on pheromone intensity; this results in a low success rate and poor energy efficiency. The UCB term enables the algorithm not only to exploit known good paths but also to explore new paths with high uncertainty. This is the foundation of the full success seen on the cost optimization (Cost-Opt) and penalty minimization (Penalty-Min) axes in the radar graph. Thanks to UCB, the algorithm distributes the load so evenly across the network that both the success rate is maximized and energy and time costs are minimized. As a result, while standard ACO exhibits a profile in the approximately 0.6–0.7 range (medium level), UCB-ACO reaches the 1.0 limit (excellent compatibility) across all axes.

In conclusion, this graph is one of the most powerful visualizations for proving the “Pareto Stability” of algorithms. The expected shape that an ideal solution should form on this graph is a smooth hexagon approaching the outer boundary on all axes; this proves that the algorithm operates without compromising any performance criterion (perfectly balancing the trade-offs).

To measure the effectiveness of the proposed UCB-ACO algorithm and the compared meta-heuristic methods under different operational conditions, 30 population sizes and 100 iterations with 10 devices and 50 tasks per device were run for 30 independent runs in three designed basic scenarios. The simulation results include best (Min/Max), worst (Max/Min), mean (Average), and standard deviation statistics for fitness, energy, delay, and success rate, which are given in Table 8, Table 9 and Table 10.

It is critical to note the statistical relationship between the evaluated average total delay (e.g., Table 8c) and the task success metric (e.g., Table 8d). An initial observation might suggest a discrepancy, as the system achieves highly optimized mean latency while simultaneously registering rejected tasks. However, this is a direct result of the framework’s inelastic constraint violation penalty (Ψ). While the average delay reflects the mean response time of valid trajectories, the success metric strictly evaluates adherence to deadlines on a per-task basis. Consequently, transient network queuing delays may cause an individual task to exceed its rigid deadline, triggering the penalty and lowering the success count, even if the overall system average latency remains low. By isolating and rejecting invalid tracks, the framework ensures that the reported average delays are not artificially skewed by failed outliers.

To validate the superiority of the proposed UCB-ACO, all algorithms were subjected to 30 independent runs with and without penalty, and the results were analyzed using the Wilcoxon rank-sum test at a 5% significance level, presented in Table 11. Table 11 summarizes the Wilcoxon rank-sum test results, while the comprehensive data for each of the 30 independent runs are provided in Table A1 and Table A2 in Appendix B.

According to Wilcoxon test results, *p* < 0.05 (especially *p* ≈ 10^−11^) was obtained, and the performance difference between the proposed UCB-ACO algorithm and competing algorithms is statistically significant. This proves that the success of the proposed method in cost minimization and constraint management is not coincidental.

In conclusion, comprehensive simulations and statistical analyses reveal that the proposed UCB-ACO based optimization framework demonstrates a significant advantage over competing algorithms in all test scenarios (balanced, energy-weighted, and time- weighted). Compared to PSO, ABO, ACO, GWO, and NSGA-II algorithms, UCB-ACO consistently achieved the lowest (best) values in both total energy consumption and network latency. Its stable performance, particularly in success rate analyses, confirms the algorithm’s ability to adapt to dynamic network conditions. Moreover, the *p*-values obtained from the Wilcoxon signed-rank test, which are far below the critical threshold value (*p* ≈ 3.0199 × 10^−11^), prove that this observed performance increase is not coincidental and is statistically highly significant. All these findings indicate that the proposed model provides both an efficient and statistically reliable optimization solution for energy- and time-constrained task-offloading applications.

## 6. Discussion

In this study, an original optimization framework integrating UCB and ACO mechanisms was developed, successfully achieving an optimal balance between energy consumption and latency in task-offloading processes within energy-constrained Wireless Sensor Networks. Comprehensive analyses showed that the proposed method exhibits superior optimization performance in terms of both operational efficiency and statistical significance compared to competing meta-heuristic algorithms under dynamic network conditions.

The superior performance of UCB-ACO relies on its ability to manage the balance between exploration and exploitation within a statistical confidence interval framework. The biggest risk encountered in traditional ACO algorithms is the trapping quickly in local optima of algorithm because of influenced by pheromone intensity. However, the UCB mechanism adds an “uncertainty bonus” to the ant colony’s decision-making process, enabling the evaluation of task offloading paths that are less frequently visited but potentially more efficient. This dynamic adaptation capability ensures that, in WSN (Wireless Sensor Network) environments where network traffic and node energy levels change in real time, the algorithm continuously updates its offloading decisions to an optimal level based on changing conditions, rather than focusing solely on past good outcomes.

The findings obtained from the proposed model make a significant contribution to the advancement of multi-objective optimization in Edge/IoT layers compared to existing approaches in the literature. The results of the study show parallelism at the objective level with the CMOEA-based approaches presented by [24], which aim to optimize energy–delay trade-offs. However, the success of the UCB1 mechanism used by [36] has been transformed into a hybrid structure with ACO in the proposed study and adapted to the specific constraints of Zigbee-based sensor networks (low bandwidth and limited energy). In addition, this study developed an optimization framework that considers the priorities of different applications by defining application-based objective functions.

The fine balance between energy consumption and delay time has a critical impact on hardware lifetime and system reliability in real-world smart home applications. For sensor nodes using low-power communication protocols such as Zigbee, minimizing energy consumption extends device battery replacement intervals from months to years, significantly reducing maintenance costs and network outages. On the other hand, keeping latency under control during task-offloading processes ensures the simultaneous and uninterrupted operation of time-sensitive tasks in smart homes, such as security systems or fire alarms. These results demonstrate how task-offloading processes can become a more sustainable digital ecosystem not only for sensors but for all resource-constrained IoT devices.

The Pareto front analysis and numerical benchmarks clearly demonstrate the statistical and comparative superiority of UCB-ACO. It is crucial to interpret these gains within the practical context of physical Fog–IoT deployments. The improvements achieved by the proposed framework translate directly into critical real-world engineering benefits:

Firstly, the persistent minimization of task energy consumption directly extends the operational lifespan of battery-constrained IoT devices. In massive-scale deployments, such as smart agriculture or industrial sensor networks, even fractional energy savings per task accumulate into months of extended battery life. So this significantly reduces the logistical costs of hardware maintenance.

Secondly, the strict adherence to latency and energy constraints—where continuous algorithms like PSO and ABO fail—has profound practical implications. In mathematical terms, constraint violations result in high penalty scores; however, in real-world Edge networks, a violated deadline equates to a dropped task or a service disruption. For mission-critical applications, such as real-time healthcare monitoring or autonomous factory robotics, ensuring deterministic task completion within strict deadlines is an absolute physical necessity. UCB-ACO’s architectural precision ensures guaranteed quality of service (QoS) and robust network reliability. It proves that its algorithmic design is not only mathematically optimal but also practically indispensable for next-generation telecommunication infrastructures. Despite the high performance exhibited by the proposed UCB-ACO model, this study has some inherent limitations. The study assumes that MDs are either stationary or moving at very low speeds. With the number of MDs set to 10, the focus was primarily on adapting the proposed optimization framework to the task-offloading problem and conducting related analyses.

The scalability of the model in much larger-scale industrial networks (IIoT) has not yet been evaluated. These limitations present a significant research gap and roadmap for expanding the developed optimization framework to cover larger-scale networks and heterogeneous network structures that include mobile devices in the future.

## 7. Conclusions and Future Work

This study proposed a context-aware UCB-ACO algorithm that optimizes energy efficiency and latency in hierarchical Fog–Edge–Cloud networks. The findings obtained confirm the potential of the proposed learning-based approach for a sustainable and efficient communication infrastructure in dynamic network ecosystems.

Task offloading is an essential strategy for IoT nodes with limited processing capacity to preserve battery life by delegating energy-intensive tasks to Edge/Fog/Cloud. In such optimization problems, a multi-objective approach to energy consumption and processing time (energy-time) is essential to balance the “conflicting objectives” where an improvement in one parameter often comes at a cost in another. The main reason why traditional meta-heuristic methods alone are insufficient is that these algorithms tend to remain static against dynamic network traffic and instantaneously changing node energies, leading to them being trapped in local optima points. Adding a “learning” (UCB) layer to the system has given the algorithm the ability to learn from past experiences and test uncertain paths with a statistical confidence interval. Thanks to this learning mechanism, a dynamic balance between exploration and exploitation has been established; thus, both the convergence speed of the system has been increased and the adaptation capability to changing environmental conditions has been maximized.

Future studies are planned to investigate the performance of the UCB-ACO model under dynamic node mobility and signal fading. Furthermore, adapting this statistically significant achievement in the simulation environment to larger-scale heterogeneous networks will increase the versatility of its application. While the proposed UCB-ACO framework provides a lightweight and sample-efficient mechanism for resource-constrained Edge networks, the integration and comparative analysis with Deep Reinforcement Learning (DRL) methods represents a highly promising research trajectory. Future work will aim to benchmark the UCB-ACO architecture against advanced DRL models, such as Deep Q-Networks (DQN) and Deep Deterministic Policy Gradient (DDPG). This future study will thoroughly investigate the trade-offs between the rapid heuristic convergence of the proposed framework and the deep neural network training overheads inherent to DRL in highly dynamic offloading environments.

## Figures and Tables

**Table 1:** Comparison table of the proposed study and existing methodologies.

Study	Author (Year)	Multi-Objective (Energy + Delay + Cost)	Algorithm(s)	Architecture/Environment	Similarity to Proposed Study
[24]	Peng et al. (2021)	Yes (Energy + Delay)	CMOEAs	Edge–Cloud	Moderate (Shared objectives)
[27]	Pettorali et al. (2025)	No (Delay only)	J-NECORA	Cloud–Edge–Things	Low (Industrial focus)
[36]	Yang et al. (2022)	Yes (Energy + Delay)	UCB1 (Multi-Armed Bandit)	Maritime Edge	High (Use of UCB mechanism)
[22]	Keshavarznejad (2021)	Yes (Energy + Delay)	NSGA-II, Bees Algorithm	Cloud–Fog–MD	High (Similar architecture)
[28]	Saif et al. (2023)	Yes (Energy + Delay)	NPSO (PSO), NSGA-II	Cloud–Fog	Moderate (PSO & Apps)
[35]	Al Reshan (2023)	Yes (Time + Cost)	IFA (Firefly Algorithm)	Cloud-only	Low (Lacks multi-tier depth, different algorithm)
[23]	Ali et al. (2022)	Yes (Delay + Cost)	DNSGA-II	Fog + Cloud	Moderate-High (NSGA-II usage)
[29]	Liu et al. (2025)	Yes (Delay + Cost)	DFGAN-LSMOEA	Terminal + Edge + Cloud	High (Architectural similarity)
[34]	Devanathan (2025)	Partially (Control-oriented)	PSO	Edge + Cloud	Low (Control oriented problem)
[30]	Huang et al. (2019)	Yes (Energy + Delay)	DDLO (Deep Learning)	WD + Edge + Cloud	Moderate-High (Multi-tier/Multi-user)
[31]	Tank & Gandhi (2023)	No (Survey)	Comparative Review	Cloud–Edge–Fog	Low (Conceptual/Review)
[32]	Dolui & Datta (2017)	No (Review)	Taxonomy & Decision Tree	Cloudlet–Fog–MEC	Low (Decision framework)
[26]	Du et al. (2018)	Yes (Energy + Delay)	Semidefinite Relaxation	Mixed Fog–Cloud	Moderate (Optimization focus)
[33]	Dinh et al. (2017)	Yes (Energy + Delay)	SDR-based Optimization	MD-to-Multiple Edge	Moderate (Task allocation focus)
[25]	Mukherjee (2022)	Yes (Delay/Price)	Wardrop Equilibrium	Edge–Cloud	Low (Economic/Pricing)
[21]	Malti et al. (2024)	Yes (Time + Energy)	Hybrid Meta-heuristic	Cloud-only	Moderate (Hybridization logic)

**Table 2:** The weights based on the application type.

Application Profile	$w_{1}$ (Latency)	$w_{2}$ (Energy)	Priority
Healthcare/Autonomous	0.8	0.2	Time-Weighted
Surveillance/Smart City	0.5	0.5	Balanced
Agriculture/Environment	0.1	0.9	Energy-Weighted

**Table 3:** Sensitivity analysis of the UCB exploration constant (c).

UCB Exploration Constant (c)	Algorithmic Behavior	Impact on Mean Fitness	Convergence Stability
c=1.0	High Exploitation	$1.1530\times 10^{6}$ $\left(+0.43\%\;\mathrm{variance}\right)$	Rapid initial convergence, prone to local optima
c=2.0 (Proposed)	Balanced	$1.1480\times 10^{6}$ $\left(\mathrm{Optimal}\;\mathrm{baseline}\right)$	Optimal balance, strictly stable convergence
c=4.0	High Exploration	$1.1514\times 10^{6}$ $\left(+0.29\%\;\mathrm{variance}\right)$	Slower convergence, broader search space coverage

**Table 4:** Hyperparameter settings of the algorithms.

Algorithm	Hyperparameters
Common Settings	Population Size = 30; maximum Iterations = 100; runs = 30
UCB-ACO	Pheromone evaporation rate (ρ)=0.1; UCB exploration constant (c) = 2.0
PSO	$\mathrm{Inertia}\;\mathrm{weight}\;(w)=0.5;\;c_{1}=1.5,$
GWO	Control parameter a linearly decreases from 2 to 0
NSGA-II	Crossover rate = 0; mutation rate = 1/N; selection = truncation (top 50%)
Discrete ABO	Modification = 1 dimension/iteration; acceptance = greedy; limit parameter = N/A
ACO	α=1;β=1; evaporation=0.1; c=2

**Table 5:** Theoretical computational complexity profiles of benchmarked algorithms.

Algorithm	Computational Complexity (Big-O Notation)	Key Drivers/Execution Bottlenecks
PSO	$O\left(T\cdot N\cdot M\right)$	Velocity and position updates for each particle per task.
GWO	$O\left(T\cdot N\cdot M\right)$	Hunting coefficient updates and distance calculations from α,β,δ wolves.
ABO	$O\left(T\cdot N\cdot M\right)$	Neighborhood search and nectar source evaluations via the fitness function.
ACO	$O\left(T\cdot N\cdot M\cdot K\right)$	Pheromone tracking and probabilistic path selection across K tiers.
NSGA-II	$O\left(T\cdot N^{2}\cdot M\right)$	$\mathrm{Dominance}\;\mathrm{sorting}\;\mathrm{and}\;\mathrm{crowding}\;\mathrm{distance}\;\mathrm{calculations}\;(N^{2}$ scaling).
UCB-ACO (Proposed)	$O\left(T\cdot N\cdot M\cdot K\right)$	$\mathrm{Lightweight}\;\mathrm{UCB}\;\mathrm{calculation}\;(O\left(M\cdot K\right)$) combined with standard ACO path construction.

**Table 6:** The simulation parameters of Algorithm and Network categories (network, device, and Zigbee protocol configurations).

Category	Parameter	Value	Category	Parameter	Value
Algorithm Parameters	Maximum Iterations	100	Network and Communication parameters	Total shared ISM bandwidth (B)	20 MHz
	Population/Swarm Size	30		Transmission Power (P_tx_)	0.3 W
	Number of Independent Runs	30		Reception Power (P_rx_)	0.1 W
	Baseline Penalty (α)	1000		Mobile Idle Power (P_idle_)	0.05 W
	Penalty Factor (β)	500		Background Noise (σ^2^)	10^−9^

**Table 7:** The simulation parameters of Task and Edge Constraints category.

Category	Parameter	Value
Task and Edge Constraints parameters	Number of Devices	10
	Tasks per Device	50
	Number of Fog Servers	3
	Number of Edge Servers	2
	Number of Cloud Servers	1
	Task Size Range	Uniformly distributed between [10, 100] MB
	Required CPU Cycles	Uniformly distributed between [1.0, 5.0] Mega cycles
	Task Deadline Threshold	Uniformly distributed between [0.1, 0.5] s
	Task Overhead Time	0.005 s
	Allocated Offloading Energy Budget	500 Joules

**Table 8:** (**a**) Statistical performance summary (fitness value metrics) in energy-weighted scenario. (**b**) Statistical performance summary (total energy (Joules)) in energy-weighted scenario. (**c**) Statistical performance summary (total delay (seconds)) in energy-weighted scenario. (**d**) Statistical performance summary (successful tasks per device) in energy-weighted scenario.

Algorithm(s)	Best (Min)	Worst (Max)	Mean	Std. Dev.
**(a)**				
PSO	1,332,849.8524	1,400,711.2384	1,365,914.9483	14,886.5108
ABO	1,312,069.0084	1,372,170.8591	1,347,457.5758	14,048.0968
ACO	1,313,777.6195	1,376,535.0784	1,344,931.6802	14,819.3849
GWO	1,141,343.0034	1,195,523.8910	1,171,431.8998	12,682.7540
NSGA-II	1,298,762.0833	1,358,395.3508	1,330,627.0282	14,413.7625
**UCB-ACO**	**1,127,771.6663**	**1,178,487.0061**	**1,151,784.1847**	**12,729.9638**
**(b)**				
PSO	279.3213	302.6806	289.0563	5.7942
ABO	274.3499	293.2167	284.1093	4.7783
ACO	272.4518	292.0488	283.5717	4.8526
GWO	220.7608	237.4790	228.7871	3.8016
NSGA-II	268.5632	289.1706	277.8774	4.9411
**UCB-ACO**	**212.7131**	**228.1925**	**220.1271**	**3.6132**
**(c)**				
PSO	1031.4653	1117.3847	1066.7834	21.2369
ABO	1012.7763	1081.4607	1048.3205	17.5135
ACO	1004.8599	1077.0354	1046.2725	17.8112
GWO	814.1911	875.0682	843.3933	13.8659
NSGA-II	990.4396	1066.3365	1024.7403	18.1031
**UCB-ACO**	**784.9930**	**841.5220**	**812.0732**	**13.1943**
**(d)**				
PSO	1.8000	0.0000	0.7133	0.4569
ABO	1.6000	0.0000	0.6400	0.4182
ACO	1.4000	0.0000	0.6400	0.3616
GWO	3.0000	0.2000	1.2800	0.6359
NSGA-II	2.0000	0.0000	0.7533	0.4508
**UCB-ACO**	**3.0000**	**0.4000**	**1.3467**	**0.6474**

Bold values indicate the results of the proposed UCB-ACO framework.

**Table 9:** (**a**) Statistical performance summary (fitness value metrics) in time-weighted scenario. (**b**) Statistical performance summary (total energy (Joules)) in time-weighted scenario. (**c**) Statistical performance summary (total delay (seconds)) in time-weighted scenario. (**d**) Statistical performance summary (successful tasks per device) in time-weighted scenario.

Algorithm(s)	Best (Min)	Worst (Max)	Mean	Std. Dev.
**(a)**				
PSO	1,324,808.24	1,408,660.41	1,361,631.38	19,512.20
ABO	1,312,391.18	1,381,801.27	1,337,782.16	18,751.43
ACO	1,307,828.37	1,378,505.41	1,340,126.38	17,520.34
GWO	1,138,989.92	1,206,916.11	1,165,892.21	16,948.61
NSGA-II	1,297,257.09	1,362,352.28	1,323,695.65	19,315.02
**UCB-ACO**	**1,121,152.91**	**1,186,968.19**	**1,147,210.40**	**16,378.69**
**(b)**				
PSO	272.5593	307.2315	287.7432	7.7311
ABO	270.6022	294.6465	280.8802	6.1585
ACO	270.4675	298.8739	281.9589	6.7912
GWO	218.3311	238.4936	226.9445	5.2436
NSGA-II	264.7764	288.8577	275.2454	6.4873
**UCB-ACO**	**210.1263**	**228.5350**	**218.3926**	**4.7490**
**(c)**				
PSO	1006.3538	1134.1845	1062.2257	28.5343
ABO	998.4789	1087.0043	1036.3495	22.6321
ACO	999.0486	1102.1738	1040.3289	24.8132
GWO	805.1456	878.7116	836.6550	19.1188
NSGA-II	976.8444	1065.3603	1015.0930	23.7476
**UCB-ACO**	**775.5504**	**842.7607**	**805.7431**	**17.3425**
**(d)**				
PSO	1.2000	0.2000	0.6733	0.3216
ABO	1.2000	0.2000	0.6533	0.3148
ACO	1.6000	0.0000	0.7333	0.3907
GWO	2.4000	0.6000	1.2733	0.4996
NSGA-II	1.4000	0.2000	0.7533	0.3137
**UCB-ACO**	**2.4000**	**0.6000**	**1.3267**	**0.4884**

Bold values indicate the results of the proposed UCB-ACO framework.

**Table 10:** (**a**) Statistical performance summary (fitness value metrics) in balanced scenario. (**b**) Statistical performance summary (total energy (Joules)) in balanced scenario. (**c**) Statistical performance summary (total delay (seconds)) in balanced scenario. (**d**) Statistical performance summary (successful tasks per device) in balanced scenario.

Algorithm(s)	Best (Min)	Worst (Max)	Mean	Std. Dev.
**(a)**				
PSO	1,322,572.1288	1,400,150.7440	1,360,796.8086	19,341.7152
ABO	1,299,908.6207	1,376,581.3601	1,339,642.4675	17,696.3729
ACO	1,297,845.0381	1,380,677.2940	1,341,608.9311	19,521.2277
GWO	1,131,876.3476	1,194,990.0915	1,167,027.2715	18,167.7213
NSGA-II	1,288,901.0933	1,358,503.2009	1,324,609.0434	17,913.5742
**UCB-ACO**	**1,110,352.4746**	**1,183,615.9960**	**1,148,343.4743**	**17,863.5462**
**(b)**				
PSO	277.8075	299.9399	288.0841	6.2873
ABO	270.5041	293.0884	281.6708	5.7685
ACO	269.5528	298.1982	282.2504	6.0259
GWO	218.9433	239.7172	227.4114	5.0293
NSGA-II	266.0681	290.6273	276.4986	5.5602
**UCB-ACO**	**210.4360**	**230.6609**	**218.8769**	**4.6177**
**(c)**				
PSO	1025.6615	1106.3615	1063.4058	22.9457
ABO	998.1508	1081.0190	1039.3365	21.1672
ACO	994.8116	1099.8467	1041.2785	22.0816
GWO	807.5050	883.3571	838.3695	18.3623
NSGA-II	981.3829	1071.3243	1019.7213	20.2506
**UCB-ACO**	**776.7175**	**850.5650**	**807.5143**	**16.8627**
**(d)**				
PSO	1.4000	0.0000	0.6333	0.3407
ABO	1.2000	0.2000	0.7000	0.2665
ACO	1.6000	0.2000	0.7000	0.3184
GWO	2.0000	0.4000	1.3467	0.4232
NSGA-II	1.6000	0.2000	0.7867	0.3928
**UCB-ACO**	**2.4000**	**0.4000**	**1.3800**	**0.4619**

Bold values indicate the results of the proposed UCB-ACO framework.

**Table 11:** Wilcoxon test results.

Algorithm(s)	*p*-Value (With Penalty)	Significance (With Penalty)	*p*-Value (Penalty-Free)	Significance (Penalty-Free)
PSO	3.0199 × 10^−11^ *	Significant	3.0199 × 10^−11^ *	Significant
ABO	3.0199 × 10^−11^ *	Significant	3.0199 × 10^−11^ *	Significant
ACO	3.0199 × 10^−11^ *	Significant	3.0199 × 10^−11^ *	Significant
GWO	8.6634 × 10^−5^	Significant	7.6950 × 10^−8^	Significant
NSGA-II	3.0199 × 10^−11^ *	Significant	3.0199 × 10^−11^ *	Significant

* Note: The identical minimum *p*-value of $3.0199\times 10^{-11}$ across multiple algorithms represents the exact asymptotic output of the Wilcoxon rank-sum test indicating complete data separation (U=0, zero overlap) between the 30 runs.

## Data Availability

The original contributions presented in this study are included in the article material. Further inquiries can be directed to the corresponding author.

## Appendix A. Physical Layer and Wireless Channel Model

To establish the physical validation baseline for the multi-tier resource allocation problem without complicating the core meta-heuristic model description, this appendix details the standard wireless channel characteristics. The achievable data transfer rate ($R_{i,j}$) for offloading a task from local device i to tier node j is calculated using the Shannon–Hartley theorem [55,56]:

(A1) $$R_{i,j}=B_{j}{log}_{2}\left(1+\frac{P_{tx}\cdot h_{i,j}}{\sigma^{2}+I}\right)$$

where

- $B_{j}$ represents the transmission bandwidth that varies dynamically depending on the selected target tier j;
- $h_{i,j}$ denotes the channel gain between the entities, modeled following a standard Rayleigh fading distribution to capture real-world environmental attenuation;
- $\sigma^{2}$ defines the background additive white Gaussian noise (AWGN) power;
- I represents the co-channel interference factor present in dense multi-user deployments.

Accordingly, the Signal-to-Interference-to-Noise Ratio (SINR), which dictates the definitive signal quality in real-world decentralized network environments [55,56], is mathematically characterized as

(A2) $$SINR=\frac{S}{I+\sigma^{2}}$$

where S represents the received signal power at the target destination node.

## Appendix B. Statistical Validation

To validate the superiority of the proposed UCB-ACO, all algorithms were subjected to 30 independent runs, both with and without penalties, and all results, including “Mean”, “Standard Deviation”, and “Best” values for each run, are presented in Table A1 and Table A2.

**Table A1:** Wilcoxon test results—with penalty scenario. Wilcoxon test result (PSO vs. UCB-ACO): *p* = 3.019 × 10^−11^, h = 1 (statistically significant).

Run No	PSO	ABO	ACO	GWO	NSGA-II	UCB-ACO
1	1.3734 × 10^6^	1.3425 × 10^6^	1.3535 × 10^6^	1.1788 × 10^6^	1.3323 × 10^6^	1.1558 × 10^6^
2	1.3601 × 10^6^	1.3310 × 10^6^	1.3394 × 10^6^	1.1664 × 10^6^	1.3094 × 10^6^	1.1461 × 10^6^
3	1.3668 × 10^6^	1.3342 × 10^6^	1.3383 × 10^6^	1.1629 × 10^6^	1.3170 × 10^6^	1.1422 × 10^6^
4	1.3423 × 10^6^	1.3402 × 10^6^	1.3380 × 10^6^	1.1617 × 10^6^	1.3131 × 10^6^	1.1380 × 10^6^
5	1.3583 × 10^6^	1.3310 × 10^6^	1.3329 × 10^6^	1.1634 × 10^6^	1.3183 × 10^6^	1.1355 × 10^6^
6	1.3720 × 10^6^	1.3711 × 10^6^	1.3752 × 10^6^	1.2006 × 10^6^	1.3605 × 10^6^	1.1740 × 10^6^
7	1.3889 × 10^6^	1.3592 × 10^6^	1.3580 × 10^6^	1.1912 × 10^6^	1.3368 × 10^6^	1.1615 × 10^6^
8	1.3601 × 10^6^	1.3639 × 10^6^	1.3556 × 10^6^	1.1841 × 10^6^	1.3277 × 10^6^	1.1602 × 10^6^
9	1.3396 × 10^6^	1.3197 × 10^6^	1.3264 × 10^6^	1.1503 × 10^6^	1.3057 × 10^6^	1.1378 × 10^6^
10	1.3595 × 10^6^	1.3468 × 10^6^	1.3348 × 10^6^	1.1728 × 10^6^	1.3229 × 10^6^	1.1557 × 10^6^
11	1.3668 × 10^6^	1.3507 × 10^6^	1.3476 × 10^6^	1.1686 × 10^6^	1.3406 × 10^6^	1.1546 × 10^6^
12	1.3500 × 10^6^	1.3260 × 10^6^	1.3334 × 10^6^	1.1658 × 10^6^	1.3129 × 10^6^	1.1378 × 10^6^
13	1.3244 × 10^6^	1.3122 × 10^6^	1.3097 × 10^6^	1.1369 × 10^6^	1.2994 × 10^6^	1.1217 × 10^6^
14	1.3688 × 10^6^	1.3587 × 10^6^	1.3544 × 10^6^	1.1804 × 10^6^	1.3177 × 10^6^	1.1630 × 10^6^
15	1.3680 × 10^6^	1.3568 × 10^6^	1.3637 × 10^6^	1.1788 × 10^6^	1.3460 × 10^6^	1.1672 × 10^6^
16	1.3401 × 10^6^	1.3251 × 10^6^	1.3218 × 10^6^	1.1536 × 10^6^	1.3154 × 10^6^	1.1411 × 10^6^
17	1.3663 × 10^6^	1.3556 × 10^6^	1.3517 × 10^6^	1.1759 × 10^6^	1.3428 × 10^6^	1.1612 × 10^6^
18	1.3493 × 10^6^	1.3100 × 10^6^	1.3195 × 10^6^	1.1421 × 10^6^	1.3069 × 10^6^	1.1220 × 10^6^
19	1.3021 × 10^6^	1.2828 × 10^6^	1.2785 × 10^6^	1.1133 × 10^6^	1.2595 × 10^6^	1.0887 × 10^6^
20	1.3496 × 10^6^	1.3235 × 10^6^	1.3299 × 10^6^	1.1395 × 10^6^	1.3111 × 10^6^	1.1238 × 10^6^
21	1.3909 × 10^6^	1.3539 × 10^6^	1.3636 × 10^6^	1.1889 × 10^6^	1.3402 × 10^6^	1.1719 × 10^6^
22	1.3851 × 10^6^	1.3658 × 10^6^	1.3561 × 10^6^	1.1919 × 10^6^	1.3476 × 10^6^	1.1601 × 10^6^
23	1.3677 × 10^6^	1.3457 × 10^6^	1.3463 × 10^6^	1.1653 × 10^6^	1.3193 × 10^6^	1.1452 × 10^6^
24	1.3474 × 10^6^	1.3272 × 10^6^	1.3373 × 10^6^	1.1623 × 10^6^	1.3174 × 10^6^	1.1432 × 10^6^
25	1.3620 × 10^6^	1.3390 × 10^6^	1.3353 × 10^6^	1.1571 × 10^6^	1.3174 × 10^6^	1.1452 × 10^6^
26	1.3674 × 10^6^	1.3484 × 10^6^	1.3417 × 10^6^	1.1735 × 10^6^	1.3325 × 10^6^	1.1476 × 10^6^
27	1.3691 × 10^6^	1.3478 × 10^6^	1.3486 × 10^6^	1.1676 × 10^6^	1.3290 × 10^6^	1.1494 × 10^6^
28	1.3466 × 10^6^	1.3084 × 10^6^	1.3105 × 10^6^	1.1487 × 10^6^	1.3126 × 10^6^	1.1322 × 10^6^
29	1.3707 × 10^6^	1.3548 × 10^6^	1.3419 × 10^6^	1.1634 × 10^6^	1.3341 × 10^6^	1.1495 × 10^6^
30	1.3658 × 10^6^	1.3455 × 10^6^	1.3479 × 10^6^	1.1723 × 10^6^	1.3266 × 10^6^	1.1428 × 10^6^
MEAN	1.3593 × 10^6^	1.3392 × 10^6^	1.3397 × 10^6^	1.1659 × 10^6^	1.3224 × 10^6^	1.1458 × 10^6^
STD DEV	1.8345 × 10^4^	2.0014 × 10^4^	1.9214 × 10^4^	1.8442 × 10^4^	1.8662 × 10^4^	1.7470 × 10^4^
BEST	1.3021 × 10^6^	1.2828 × 10^6^	1.2785 × 10^6^	1.1133 × 10^6^	1.2595 × 10^6^	1.0887 × 10^6^

**Table A2:** Wilcoxon test results—penalty-free scenario. Wilcoxon test result (PSO vs. UCB-ACO): *p* = 3.0199 × 10^−11^, h = 1 (statistically significant).

Run No	PSO	ABO	ACO	GWO	NSGA-II	UCB-ACO
1	996.9389	975.5487	990.1734	791.1781	964.7050	760.5960
2	982.6983	947.9322	945.9737	773.5182	925.4727	741.7333
3	986.3728	953.8377	945.2668	770.3836	930.3306	743.4539
4	958.4001	957.9446	958.4804	768.4844	924.1303	739.7597
5	996.7396	957.3826	960.6533	776.9590	944.2143	745.9909
6	997.6046	992.0178	991.5790	803.8769	978.6657	771.1500
7	1.0120 × 10^3^	980.1317	984.2309	792.1204	954.2794	760.0173
8	996.2251	1.0000 × 10^3^	985.4532	805.4885	954.3796	769.9884
9	959.4940	947.1648	942.9705	760.2149	928.7951	733.9675
10	982.2004	966.3568	973.7182	784.0629	945.9761	751.9862
11	989.7349	960.3385	960.7945	769.2094	949.0321	746.6212
12	972.6890	948.1390	958.1756	774.9525	929.0108	742.6604
13	939.4437	945.3489	938.0361	749.8244	926.8317	726.4743
14	991.0429	993.3129	985.9692	797.0695	942.7986	764.6077
15	985.8967	986.4633	992.7889	791.1412	972.5439	764.4931
16	974.5326	953.4771	955.2973	765.0864	942.8128	736.4340
17	997.8711	981.2950	981.9287	786.1683	966.9225	756.3635
18	970.3263	922.9963	936.0356	759.8556	933.2423	732.5258
19	912.1148	904.2576	908.3866	731.6888	877.8232	696.7215
20	982.1370	940.2471	951.1952	758.7193	934.1692	738.4820
21	1.0192 × 10^3^	979.4640	996.2816	801.3156	964.5414	770.6028
22	1.0135 × 10^3^	988.6135	991.1109	806.1801	970.9320	769.6812
23	992.4489	959.3218	973.0105	769.6325	930.8447	743.5213
24	971.7393	949.1161	953.4417	767.6727	932.0349	739.7558
25	972.9982	957.5228	958.0376	767.6765	928.6391	744.1675
26	986.4249	976.2263	970.6125	786.7616	950.9954	754.6415
27	982.9131	979.0629	976.9576	777.0983	944.1524	754.3637
28	964.9506	938.1744	937.4755	759.3774	934.9630	732.1487
29	985.8634	978.9233	949.5561	775.1551	949.6239	748.0906
30	982.2271	969.0658	980.4937	778.1713	946.0938	749.9805
MEAN	981.8938	962.9908	964.4695	776.6348	942.6319	747.7007
STD DEV	21.3788	21.8150	21.5196	17.4342	19.7339	15.8573
BEST	912.1148	904.2576	908.3866	731.6888	877.8232	696.7215
